# Primate‐Specific DAZ Regulates Translation of Cell Proliferation‐Related mRNAs and is Essential for Maintenance of Spermatogonia

**DOI:** 10.1002/advs.202400692

**Published:** 2024-05-23

**Authors:** Ningjing Ou, Yuci Wang, Shuai Xu, Jiaqiang Luo, Chenwang Zhang, Yangyi Zhang, Xiaoyan Shi, Minggang Xiong, Liangyu Zhao, Zhiyong Ji, Yuxiang Zhang, Jingpeng Zhao, Haowei Bai, Ruhui Tian, Peng Li, Erlei Zhi, Yuhua Huang, Wei Chen, Ruiqi Wang, Yuxuan Jin, Dian Wang, Zheng Li, Hao Chen, Chencheng Yao

**Affiliations:** ^1^ Department of Andrology Center for Men's Health Urologic Medical Center Shanghai General Hospital Shanghai Jiao Tong University School of Medicine Shanghai 200011 China; ^2^ Department of Urology Department of Interventional Medicine Guangdong Provincial Key Laboratory of Biomedical Imaging The Fifth Affiliated Hospital Sun Yat‐sen University Zhuhai Guangdong 519000 China; ^3^ Department of Human Cell Biology and Genetics Joint Laboratory of Guangdong & Hong Kong Universities for Vascular Homeostasis and Diseases School of Medicine Southern University of Science and Technology Shenzhen Guangdong 518000 China

**Keywords:** DAZ, microdeletion of AZFc, proliferation, RNA binding protein, spermatogenic failure

## Abstract

Primate‐specific *DAZ* (deleted in azoospermia) has evolved in the azoospermia factor c (AZFc) locus on the Y chromosome. Loss of *DAZ* is associated with azoospermia in patients with deletion of the AZFc region (AZFc_del). However, the molecular mechanisms of *DAZ* in spermatogenesis remain uncertain. In this study, the molecular mechanism of *DAZ* is identified, which is unknown since it is identified 40 years ago because of the lack of a suitable model. Using clinical samples and cell models, it is shown that *DAZ* plays an important role in spermatogenesis and that loss of *DAZ* is associated with defective proliferation of c‐KIT‐positive spermatogonia in patients with AZFc_del. Mechanistically, it is shown that knockdown of *DAZ* significantly downregulated global translation and subsequently decreased cell proliferation. Furthermore, DAZ interacted with PABPC1 via the DAZ repeat domain to regulate global translation. DAZ targeted mRNAs that are involved in cell proliferation and cell cycle phase transition. These findings indicate that DAZ is a master translational regulator and essential for the maintenance of spermatogonia. Loss of *DAZ* may result in defective proliferation of c‐KIT‐positive spermatogonia and spermatogenic failure.

## Graphical abstract

It is revealed that DAZ interacts with PABPC1 via the DAZ repeat domain to regulate the translation of target transcripts involved in cell proliferation and cell cycle phase transition (such as c‐*KIT* and SMC2) in the human testis. Loss of *DAZ* (Y chromosome AZFc deletion) may result in defective proliferation of c‐KIT‐positive spermatogonia and spermatogenic failure.

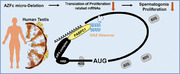

## 1 Introduction

Microdeletions on the Y chromosome are the most common genetic etiology for spermatogenic failure.^[^ ^1^ ^]^ The prevalence of microdeletions on the Y chromosome is 2–10% in infertile men with severe oligozoospermia (<5 million sperm/mL ejaculate) or azoospermia.^[^ ^2^, ^3^ ^]^ The Y chromosome contains several genes in the azoospermia factor (AZF) region that are essential for spermatogenesis.^[^ ^4^ ^]^ The AZF region is divided into AZFa, AZFb, and AZFc based on histological characteristics.^[^ ^5^ ^]^ The most prevalent type of AZF deletion is AZFc_del, which accounts for ≈80% of cases.^[^ ^6^ ^]^ The pathological phenotypes of AZFc_del are heterogeneous and include Sertoli cell‐only syndrome, maturation arrest, and hypospermatogenesis.^[^ ^4^, ^7^, ^8^ ^]^ However, the precise pathological molecular mechanisms underlying the phenotypes resulting from AZFc_del are unknown.

Primate‐specific *DAZ* (deleted in azoospermia) is assumed to be the most important gene in the AZFc region.^[^ ^9^, ^10^ ^]^ There are four copies of *DAZ* in this region and deletion of the entire AZFc region results in the loss of these copies and spermatogenic failure.^[^ ^11^ ^]^ *DAZ* is expressed predominantly in the testis and has been found in Catarrhini (Old World monkeys, apes, and humans) but not in New World monkeys or mice.^[^ ^9^, ^12^, ^13^ ^]^ The role of DAZ in spermatogenesis and the exact molecular mechanisms involved remain unclear because the function of DAZ cannot be evaluated in a mouse model. Additional evidence for the role of DAZ in spermatogenesis has been derived from functional studies of *Dazl* (Deleted in Azoospermia‐Like), the autosomal homolog of DAZ. Knockout of *Dazl* in mice leads to severely impaired development of germ cells and infertility in both sexes.^[^ ^14^ ^]^

DAZ family proteins, including DAZ, DAZL, and BOLL, contain N‐terminus RNA recognition motif (RRM) domains and C‐terminus DAZ repeats. Previous studies have demonstrated that DAZL plays a critical role in regulating the translation and stability of target mRNAs at three stages of spermatogenesis (spermatogonia, spermatocyte, and spermatid).^[^ ^15^, ^16^, ^17^, ^18^ ^]^ BOLL have been reported to play a role in the regulation of cell cycle phase transition during meiosis in mice.^[^ ^19^ ^]^ However, BOLL was found to be expressed strictly at the spermatocyte stage.^[^ ^20^ ^]^ The expression of DAZL and BOLL in the stages of the cycle of seminiferous tubules and their target mRNAs were different, suggesting that they also have different functions in spermatogenesis.^[^ ^16^ ^]^ Interestingly, spermatogenesis in *Dazl* knockout mice can be partially rescued to different degrees by human DAZL or DAZ.^[^ ^21^, ^22^ ^]^ These findings suggest that human DAZ and DAZL proteins may play different roles in spermatogenesis. Furthermore, limited homology was identified between human DAZ and mouse DAZL.^[^ ^23^ ^]^ DAZL contains only one RRM domain and one DAZ repeat domain whereas DAZ1 contains three RRM domains and nine DAZ repeat domains. DAZ2 and DAZ3 each have one RRM domain and DAZ4 contains two RRM domains.^[^ ^24^ ^]^ It is not clear whether or not multiple copies of the RRM and DAZ repeat domain in DAZ function differently from the single domain in DAZL. Deletion of *DAZ* is associated with spermatogenesis disorder in males, but its expression in specific stages of spermatogenesis and the molecular mechanisms involved still remain unclear.

The aims of this study were to identify the key gene in the AZFc region and to explore its molecular function. Single‐cell RNA sequencing (scRNA‐seq) data for the testis from patients with AZFc_del revealed distinct transcriptomics in the spermatogonia stage, in which *DAZ* was highly expressed. We also confirmed that DAZ was highly expressed in this stage, whereas DAZL was mainly expressed in spermatocytes in the human testis. These findings suggest that a deficiency of *DAZ* may be responsible for the defects in spermatogonia in patients with AZFc_del. Furthermore, biochemical and cell investigations revealed that target transcripts recognized by the RRM domain of DAZ are involved in proliferation and cell cycle phase transition, while the C‐terminus DAZ repeat domain interacts with polyadenylate binding protein cytoplasmic 1 (PABPC1) to promote translation of the target mRNAs. Collectively, our findings indicate that AZFc_del decreases the proliferation of spermatogonia, leading to severe oligozoospermia or azoospermia.

## 2 Result

### 2.1 Single‐Cell RNA‐Seq Profiling and IF Staining Demonstrated That Deficiency of *DAZ* is Associated with Spermatogenic Failure

**Figure 1:**
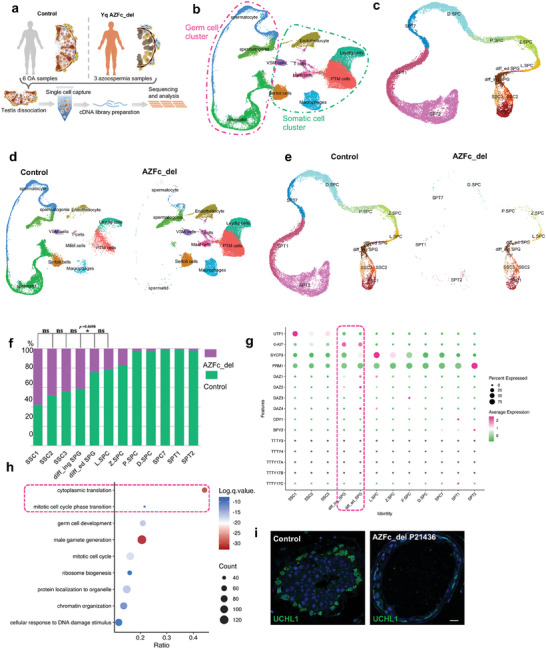
Testicular transcriptome characteristics in spermatogenic failure affected patients with AZFc_del via scRNA‐seq. a) Schematic illustration of the scRNA‐seq experimental workflow. b) Cell types colored in UMAP plots of all testicular cells from three cases with AZFc_del and six OA controls. c) UMAP plots for all germ cells from six OA controls merged with three cases of AZFc_del. d) UMAP plots for all testicular cells from six OA controls and three cases of AZFc_del, respectively. e) UMAP plots for germ cells from six OA controls and three cases of AZFc_del, respectively. f) Bar plot showed the proportion of germ cells at each stage in the AZFc_del group and OA control group. Differences in cell proportions were compared between two consecutive stages using Pearson's chi‐squared test. ^*^ *p *< 0.05. ns, not statistically significant. g) Bubble chart showing the expression levels of genes that are important in spermatogenesis and key genes in the AZFc region in human germ cells according to the scRNA sequencing data for OA controls with normal spermatogenesis. h) GO analysis of DEGs between transcriptomics of spermatogonia from OA controls and AZFc_del cases. i) Immunofluorescence detected expression of UCHL1 (a marker of spermatogonial stem cells) in human testicular tissue from AZFc_del cases and OA controls. Scale bar, 10 µm. Abbreviations: AZFc, azoospermia factor c; AZFc_del, deletion of the AZFc region; DEGs, differentially expressed genes; GO, Gene Ontology; OA, obstructive azoospermia; PTM cell, peritubular myoid cell; VSM cell, vascular smooth muscle cell; UMAP, uniform manifold approximation and projection.

The molecular mechanism of spermatogenic failure caused by AZFc_del was investigated using scRNA‐seq to explore differences in transcriptomics during spermatogenesis in the testis of patients with AZFc_del (Figure S1a, Table S4, Supporting Information). All patients with AZFc_del enrolled for scRNA‐seq were confirmed to be *DAZ*‐deficient but to have germ cells in the seminiferous tubules (Figure S1b–e, Supporting Information). The scRNA‐seq profiles for three AZFc_del patients and six OA controls with normal spermatogenesis from our previous reports^[^ ^25^ ^]^ were subjected to further analysis. A total of 64 585 single cells were divided into 11 cell clusters (including germ cell clusters and somatic clusters) (**Figure** **1**a,b). The germ cells were subdivided into 12 subclusters using an unsupervised classification method via reported marker genes (listed in Table S3, Supporting Information) for each germ cell stage^[^ ^27^, ^28^, ^29^ ^]^ (Figure 1c).

Re‐clustering of testicular cells from the three AZFc_del samples and six OA controls showed that the number of germ cells was significantly reduced in the AZFc_del group (Figure 1d,e; Figure S1f, Supporting Information). The number of c‐KIT (KIT Proto‐Oncogene)‐positive spermatogonia (diff_ed SPG), but not of spermatogonial stem cells (SSCs), was significantly lower in patients with AZFc_del than in controls (Figure 1f). Also, trajectory analysis showed that the number of c‐KIT (KIT Proto‐Oncogene)‐positive spermatogonia (diff_ed SPG), spermatocytes, and spermatids were lower in patients with AZFc_del than in controls (Figure S1g–i, Supporting Information), indicating that the germ cell number was dramatically reduced at differentiated spermatogonia (diff_ed SPG) stage. These findings suggested that genes in the AZFc region play an essential role in the c‐KIT‐positive spermatogonia stage. Intriguingly, *DAZ* was transcribed at this stage, while other genes, including *BPY2* (Basic Charge Y‐Linked 2) and *CDY1* (Chromodomain Y‐Linked 1) in the AZFc region (Figure 1g) were mostly expressed in the postmeiotic stage of spermatogenesis, suggesting that depletion of *DAZ* is responsible for deficient c‐KIT positive spermatogonia in patients with AZFc_del. To determine whether SSCs, which are the precursors of c‐KIT‐positive spermatogonia, were affected by AZFc_del, we performed IF staining of testicular sections using the SSC‐specific marker antibody known as ubiquitin carboxyl‐terminal hydrolase isozyme L1 (UCHL1; Figure 1i). Consistent with the scRNA‐seq data, UCHL1 staining showed similar quantities of SSCs in patients with AZFc_del, suggesting that SSCs are less affected in AZFc_del. However, the morphology of SSCs in patients with AZFc_del differed from that in the control group, which manifested with smaller nuclei (Figure 1i).

Next, we performed IF staining assay to examine the expression of DAZ in the human testis. We found that DAZ was strongly expressed in differentiating spermatogonia marked by c‐KIT and weakly expressed in spermatocytes in the human testis (Figure S1j, Supporting Information). However, the other two proteins in the DAZ family, DAZL and BOLL, were abundantly expressed in SYCP3‐positive spermatocytes detected at both the mRNA and protein levels, with weak expression of DAZL at the protein level in spermatogonia (Figure S1k,l, Supporting Information). Therefore, we focused on the molecular function of DAZ in c‐KIT‐positive spermatogonia. Comparison of differentially expressed genes (DEGs) in c‐KIT‐positive spermatogonia between the AZFc_del and OA control samples showed that the main enriched Gene Ontology terms were “cytoplasmic translation” and “mitotic cell cycle phase transition” (Figure 1h), suggesting that DAZ is associated with cytoplasmic translation and cell cycle phase transition. Overall, the above results indicated that depletion of *DAZ* may be the key factor leading to the deficient proliferation of spermatogonia in patients with AZFc_del.

### 2.2 Deficiency of Endogenous *DAZ* Induces Cell Cycle Interruption But Not Apoptosis

**Figure 2:**
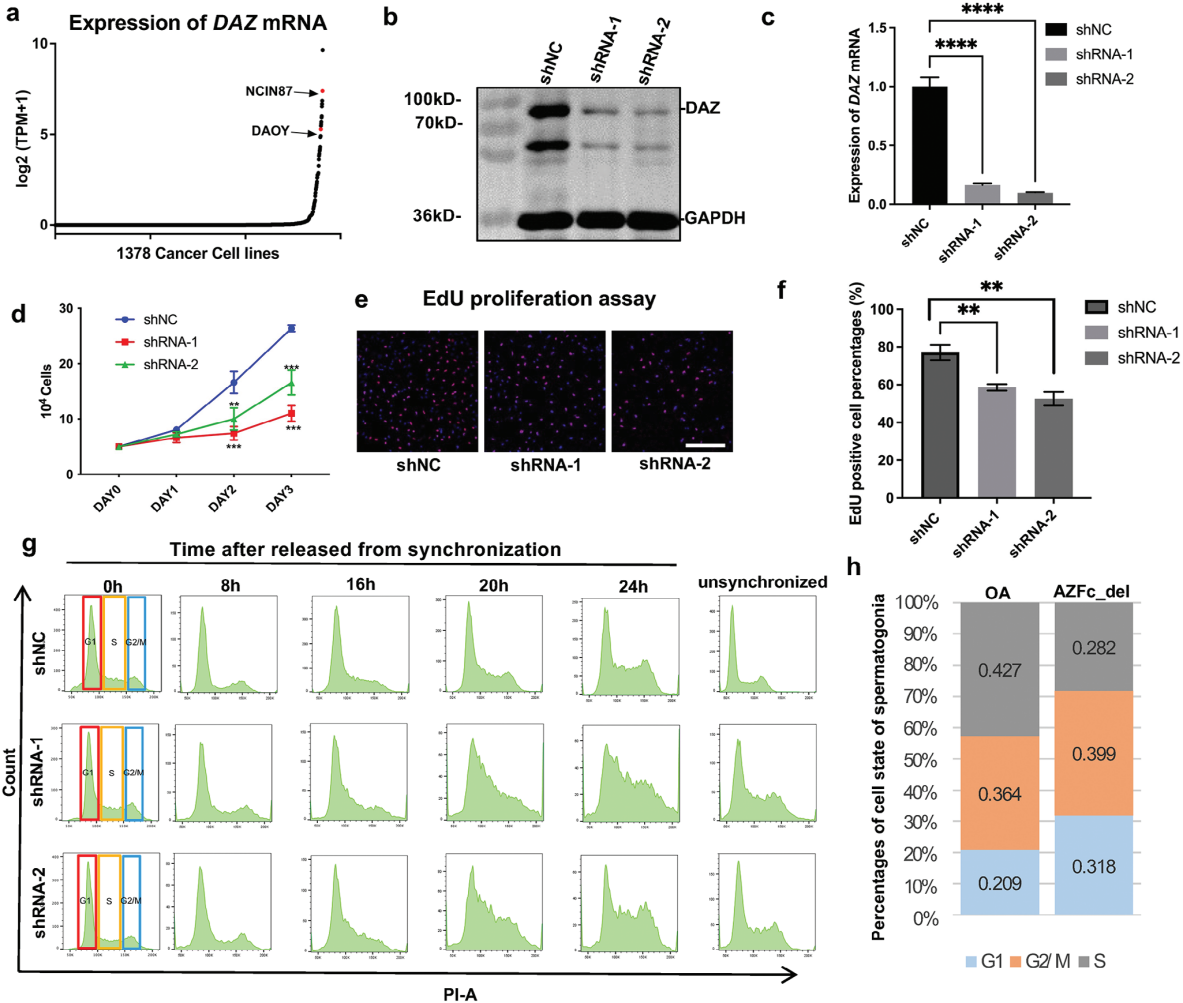
Knockdown of endogenous *DAZ* in DAOY cells interrupted the cell cycle and downregulated cell proliferation. a) Dot plot showing expression of *DAZ* mRNA in cancer cell lines based on data from Depmap. The upper red dot indicates the NCIN87 cell line and the lower red dot indicates the DAOY cell line. b) Western blotting showed expression of DAZ in DAOY cells after downregulation. c) Quantitative PCR assays showed efficient downregulation of DAZ in DAOY cells (shRNA1 and shRNA2 vs shNC using one‐way ANOVA). The data are shown as the mean ± standard deviation (*n* = 3). ^****^ *p *< 0.0001. d) A cell counting assay showed growth of *DAZ* knockdown cells (shRNA1 and shRNA2 versus shNC using one‐way ANOVA). The data are shown as the mean ± standard deviation (*n* = 3). ^**^ *p *< 0.01, ^***^ *p *< 0.001. e) EdU proliferation assay showing cell proliferation of DAOY cells after *DAZ* knockdown (shRNA1 and shRNA2 vs shNC, *n* = 3). Scale bar, 50 µm. f) Bar graph showing the proportion of EdU‐positive cells after *DAZ* knockdown (shRNA1 and shRNA2 vs shNC using Pearson's chi‐squared test). The data shown are the mean ± standard deviation (*n* = 3). ^**^ *p *< 0.01. g) Propidium iodide staining detected by flow cytometry showing the distribution of each cell cycle stage (G1, S, and G2/M phase) after *DAZ* knockdown (shRNA1 and shRNA2 vs shNC, *n* = 3) at 0, 8, 16, 20, and 24 h after serum supplementation and unsynchronized cells. h) Percentage bar graph showing cell cycle status of c‐KIT‐positive spermatogonia in OA controls and patients with AZFc_del according to cell cycle markers (data from single‐cell RNA‐seq). Abbreviations: ANOVA, analysis of variance; AZFc, azoospermia factor c; AZFc_del, deletion of the AZFc region; NC, negative control; OA, obstructive azoospermia; qPCR, quantitative polymerase chain reaction; sh, short hairpin.

Given that *DAZ* is only present in a few species of primates, there is no appropriate animal model or spermatogenic cell line for investigation of the molecular function of DAZ. Therefore, in this study, a human cancer cell database (https://depmap.org/portal/) was used to explore cell lines with high *DAZ* expression (e.g., DAOY and NCIN87 cells) (**Figure** **2**a). Western blotting and RT‐qPCR showed that DAZ was highly expressed in DAOY cells derived from a desmoplastic cerebellar medulloblastoma in a 4‐year‐old boy (Figure S2a,b, Supporting Information).^[^ ^30^ ^]^ Therefore, DAOY cell line was chosen for the investigation of the molecular function of DAZ. The role of DAZ in the fate determination in DAOY cells was investigated by RNA interference. As shown in Figure 2b,c, RT‐qPCR, and Western blotting demonstrated efficient knockdown of DAZ in DAOY cells (down to ≈20% for shRNA1 and 30% for shRNA2). Interestingly, cell proliferation was significantly decreased at 48 and 72 h after the knockdown of *DAZ* (*p *< 0.01) (Figure 2d–f). After synchronization of the cell cycle by serum starvation for 12 h, cell arrest was then released by adding 10% FBS. FACS analysis was then performed to identify cell cycle progression at 0, 8, 16, 20, and 24 h after release. As shown in Figure 2g, after synchronization for 0, 8, 16, 20, and 24 h, the cells in the control group showed a normal distribution between G1, S, and G2/M phases. However, in cells with *DAZ* knockdown, the proportion of cells in G1 phase decreased and the proportion of those in S phase increased significantly from 16 h onward. It was difficult to distinguish between cells in S phase and those in G2/M phase, indicating that multiple periods in the cell cycle were affected. Furthermore, Annexin V/PI staining and TUNEL assays found no significant change in the rate of apoptosis in DAOY cells after the knockdown of *DAZ* (*p *> 0.05) (Figure S2c–e, Supporting Information). These results are consistent with the above‐mentioned observations for the single‐cell data, which showed that the proportion of c‐KIT‐positive cells in G1/S phase was significantly smaller in patients with AZFc_del than in OA controls (Figure 2h), suggesting that DAZ is involved in regulation of cell proliferation but not apoptosis.

### 2.3 DAZ Directly Interacts with Translation Factor PABPC1 via the DAZ Repeat Domain and Regulates mRNA Translation

To determine the mechanism via which DAZ regulates cell cycle phase transition, we sought to identify the interacting partners of DAZ using a DAZ‐overexpressing line with tags for both FLAG (with the DYKDDDDK sequence) and HA (with the YPYDVPDYA sequence). In view of their similar protein sequences, antibodies made for the DAZ1 protein could bind to all four of the DAZ proteins (DAZ1, DAZ2, DAZ3, and DAZ4). Expression of endogenous DAZ proteins in DAOY cells was examined by Western blotting. The band with the anticipated molecular weight of the DAZ1 protein was the most abundant as an endogenous DAZ protein, which corresponded to expression of the DAZ protein in both DAOY cells and the human testis (Figure S2a, Supporting Information). *DAZ1* was chosen as being representative of the *DAZ* genes for examination of the molecular mechanism of these genes. The percentage of positive cells in a cell line with stable overexpression of *DAZ1* was evaluated by IF. And the results showed that more than 80% of the cells expressed exogenous DAZ (Figure S3a, Supporting Information).

**Figure 3:**
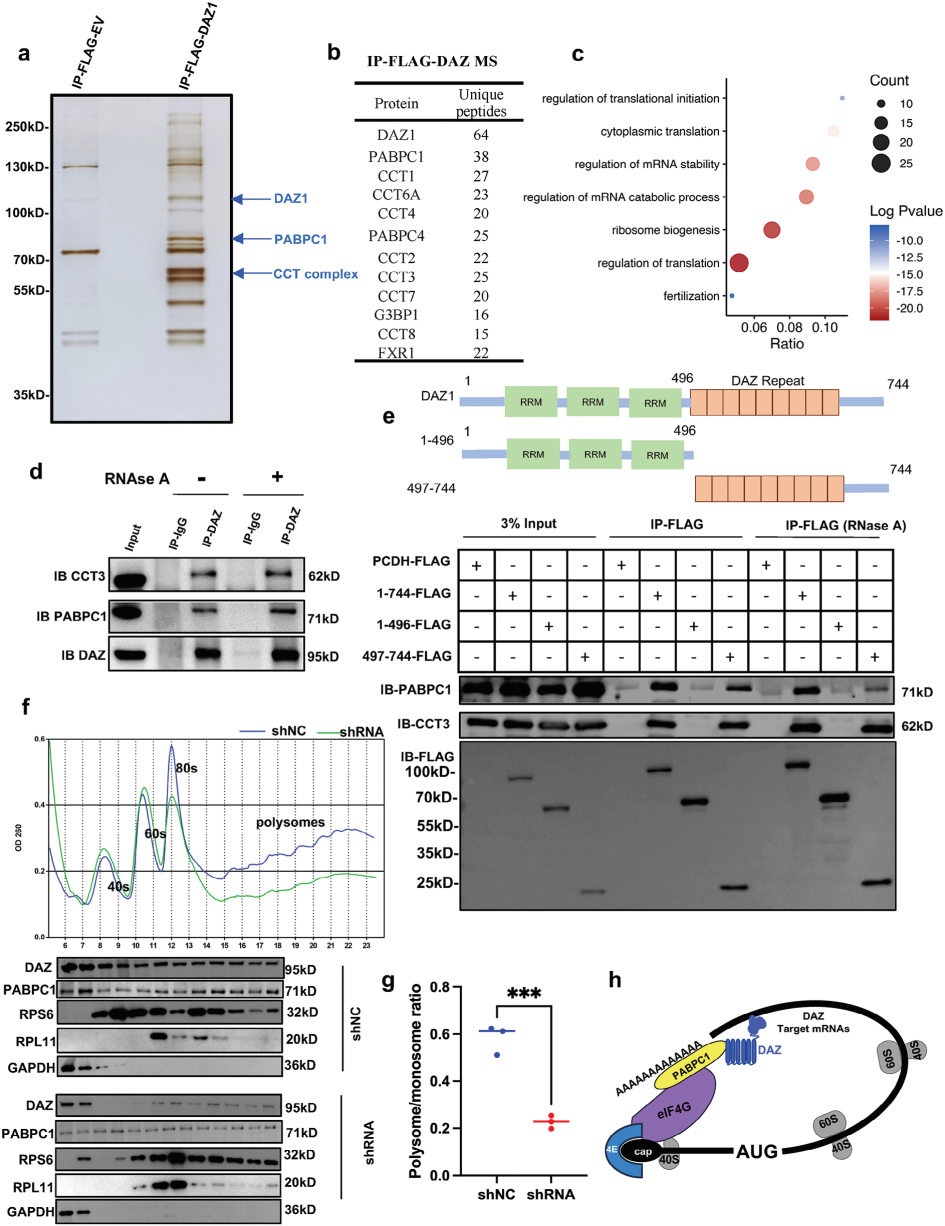
DAZ regulates global translation by interacting with translation factor PABPC1 via the DAZ repeat domain. a) Identification of DAZ‐associated partners by immunoprecipitation experiments using FLAG beads in a DAOY cell line stably overexpressing DAZ‐FLAG. Immunoprecipitated DAZ‐FLAG protein complexes were separated by sodium dodecyl sulfate‐polyacrylamide gel electrophoresis, and the gel was stained with silver stain before mass spectrometry. b) DAZ‐associated protein candidates identified by mass spectrometry. c) GO analysis of DAZ‐associated proteins identified by mass spectrometry.d) Immunoprecipitation with anti‐DAZ antibodies (with or without RNases) and Western blotting analysis with the indicated antibodies (CCT3 and PABPC1). e) Plasmid‐expressing truncations of the RRM domain (1–496 amino acids) and the DAZ repeat region (497–744 amino acids) of DAZ1 (1–744 amino acids) were transfected in HEK 293T cells. We used agarose beads conjugated with FLAG to pull the complex containing DAZ and its interaction protein by immunoprecipitation with or without RNase in the cell lysate of HEK 293T cells that were transfected with the various DAZ truncations. Western blotting showed expression of PABPC1 and CCT3 after IP using the FLAG tag of the different DAZ truncations. f) Polysome profiling showed the change of global translation of *DAZ* knockdown in DAOY cells. Western blotting of PABPC1, an interactive partner of DAZ, in different fractions after polysome profiling. Western blotting of RPS6 (a 40S ribosomal marker) and RPL11 (a 60s ribosome marker) demonstrating the components of the fraction after polysome profiling. GAPDH was assumed as a house‐keeper gene expressed in free parts of the lysate without ribosomes. PABPC1 was identified as an interactive partner of DAZ. g) Scatter plot showed the polysome/monosome ratio of DAOY cells after *DAZ* knockdown (shRNA vs shNC using one‐way ANOVA). The data are shown as the mean ± standard deviation (*n* = 3). ^***^ *p *< 0.001. h) Schematic model showing that DAZ may activate translation of its target mRNAs by binding the 3′‐UTR and interacting with components of the translation machinery (complex of PABPC1, eIF4 complex, and the cap of the mRNAs). Abbreviations: NC, negative control; sh, short hairpin; IB, immunoblotting; RPL11, 60S ribosomal protein L11; RPS6, 40S ribosomal protein S6; UTR, untranslated region; ANOVA, analysis of variance.

Immunoprecipitation (IP) coupled with mass spectrometry (MS) in DAZ1‐overexpressing (DAZ1‐OE) DAOY cells identified 103 proteins that could interact with DAZ1 (**Figure** **3**a,b; Table S5, Supporting Information). Interestingly, these proteins were significantly enriched in the cytoplasmic translation pathway (Figure 3c). The most prevalent unique peptide hits for the purified DAZ1 complex were the RNA binding protein PABPC1 (Poly A Binding Protein Cytoplasmic 1), which could interact with the 3′ poly(A) tail of eukaryotic mRNAs via RNA recognition motifs^[^ ^31^ ^]^ and the chaperonin‐containing TCP1 (CCT) complex (containing CCT1‐8).^[^ ^32^ ^]^ Furthermore, the interaction between DAZ1 and PABPC1/CCT3 (chaperonin‐containing TCP1 subunit 3) was confirmed by co‐IP (Figure 3d). Importantly, the interaction between DAZ1 and PABPC1/ CCT3 was not affected by the removal of RNA using RNase A, suggesting that their interaction is RNA‐independent (Figure 3d). Furthermore, co‐IP was performed to confirm the interaction between DAZ and other proteins identified in the mass spectrum, which were assumed to be translational regulators and included FXR1 (FMR1 Autosomal Homolog 1), FXR2 (FMR1 Autosomal Homolog 2), ELAVL1 (ELAV‐Like RNA Binding Protein 1), EIF4G1 (Eukaryotic Translation Initiation Factor 4 Gamma 1), RTRAF (RNA Transcription, Translation and Transport Factor), and G3BP1 (G3BP Stress Granule Assembly Factor 1). These proteins were detected in the DAZ1 pulldown elution without RNase A treatment but decreased significantly in the elution with RNase A (Figure S3b, Supporting Information), indicating that they interacted with DAZ in an RNA‐dependent manner.

Next, we sought to identify the domain of DAZ that mediates interaction with its partners. Plasmid‐expressing truncations carrying the RRM domain (1‐496 amino acids) and the DAZ repeat region (497–744 amino acids) of DAZ1 (1–744 amino acids) were transfected into HEK 293T cells (Figure 3e). Interactions were then evaluated using different DAZ truncations after the pull‐down of the complex by the FLAG tag. As shown in Figure 3e, the DAZ repeat domain rather than the RRM domain was necessary and sufficient for interaction with both PABPC1 and CCT3, indicating that the DAZ1 and DAZ repeat domain can interact with CCT3 (a subunit of the CCT complex) and PABPC1 with or without RNase A. This finding demonstrates that the DAZ repeat domain can mediate the interaction of DAZ1 with PABPC1 and CCT3 in an RNA‐independent manner. PABPC1 was most prevalent in the distinct peptide hits for the purified DAZ complex. In addition, DAZL, the DAZ family protein, was previously shown to control translation in germ cells.^[^ ^17^ ^]^ Therefore, we conducted additional experiments to explore the role of DAZ in regulating translation.

As described previously, binding of PABPC1 to poly(A) on mRNAs promotes recruitment of ribosomes and initiation of translation.^[^ ^33^ ^]^ Therefore, we performed polysome profiling to investigate translational changes after DAZ knockdown in DAOY and NCIN87 cells (Figure 2b; Figure S3c, Supporting Information). We found that the proportion of polysome fractions (where active translation of mRNA occurs) was significantly lower in *DAZ* knockdown cells than in control cells, indicating that DAZ regulates global translation in DAOY and NCIN87 cells (Figure 3f; Figure S3d, Supporting Information). Western blotting verified proteins (DAZ, PABPC1, RPS6, RPL11) distribution in corresponding fraction (Figure 3f). The results showed that DAZ and PABPC1 were both present in all fractions, indicating DAZ was involved in the process of translation. The measurement showed that in areas under the curve (AUC) of polysomes and monosomes the polysome/monosome (P/M) ratio was downregulated after *DAZ* knockdown in both DAOY and NCIN87 cells (Figure 3g; Figure S3e, Supporting Information). Collectively, these findings showed that DAZ interacted with PABPC1 in an RNA‐independent manner via the DAZ repeat domain, which may be involved in regulating the translation of DAZ target transcripts. A previous study reported an elegant model of eukaryotic translation initiation, in which the 3′ poly(A) tail of an mRNA, which is occupied by poly(A)‐binding proteins (PABPs), communicates with eIF4 complex (eIF4G and eIF4E) to enhance translation.^[^ ^34^, ^35^ ^]^ Collectively, we have summarized a pattern of DAZ regulation in cellular translation (Figure 3h). However, the target mRNAs recognized by DAZ need to be further characterized in order to clarify the cellular biological functions of DAZ.

### 2.4 DAZ1 Promotes Efficient Translation of Cell Cycle‐Related Transcripts via Binding to 3′‐Untranslated Regions

**Figure 4:**
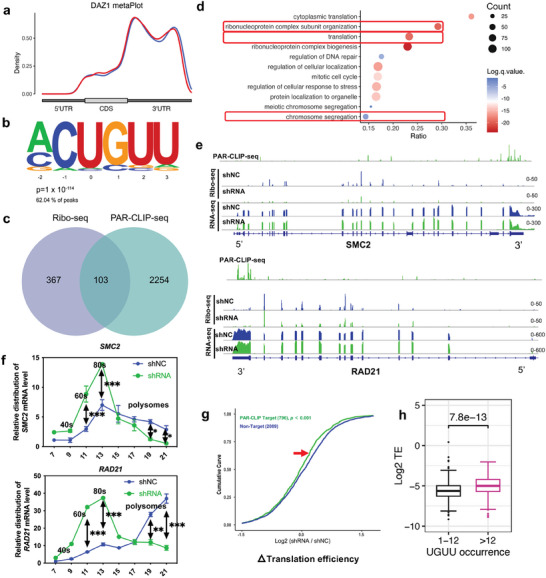
DAZ1 promotes efficient translation of cell cycle‐related targets by binding to 3′‐untranslated regions. a) Metagene analysis of PAR‐CLIP‐seq peaks of DAZ target genes along the length of the mRNA transcripts identified in two biological replicates (red and blue lines represent the different replicates). b) Discovery of a de novo motif (UGUU) from replicated DAZ peaks. DAZ‐binding motifs were identified by HOMER findMotifsGenome.pl from the PAR‐CLIP‐seq peaks of two biological replicates. The motif length was restricted to 4–8 nucleotides. The *P*‐value was calculated from the random background sequences with a ZOOPS score. c) Venn diagram showing the overlap of translation‐downregulated genes detected by Ribo‐seq and DAZ target genes detected by PAR‐CLIP‐seq. d) GO analysis of DAZ targets detected by PAR‐CLIP‐seq overlapped with the Ribo‐seq. e) Integrated Genome Viewer (IGV) showing the mapped reads of the binding peaks identified by PAR‐CLIP‐seq and ribosome‐bound fragments identified by Ribo‐seq of two DAZ targets involved in the cell cycle pathway (*SMC2* and *RAD21*) after *DAZ* knockdown. The histograms represent the sum of mapped reads along the genome. The mapped reads of RNA‐seq and Ribo‐seq were normalized by the sequencing depth. f) The broken line chart showed the relative distribution of the mRNA level of *SMC2* and *RAD21* in the different fractions (consistent with polysome profiling in Figure 3f) isolated by the sucrose gradient. The same volume (50 µL) of the fractions was loaded to perform RT‐qPCR (shRNA vs shNC using one‐way ANOVA). The data are shown as the mean ± standard deviation (*n* = 3). ^*^ *p *< 0.05; ^**^ *p *< 0.01; ^***^ *p *< 0.001. g) After integration of the Ribo‐seq with the PAR‐CLIP‐seq, protein‐coding genes were divided into RAR‐CLIP‐targeted genes and non‐PAR‐CLIP‐targeted genes. Bioinformatic analysis showed a cumulative distribution of log2‐fold changes in translation efficiency (ratio of ribosome‐bound fragments to input RNA) between shNC and shRNA. The cumulative distribution was analyzed empirically based on the value of the log2‐fold change in translation efficiency separately in R. The *P*‐value was calculated using the Wilcoxon test (two‐sided).^[^ ^43^ ^]^ The red arrow between the green and blue lines around y = 0.75 highlights the decrease in translation efficiency for DAZ target versus DAZ nontarget mRNAs. h) Boxplot showed the difference in translation efficiency between DAZ target genes with more than 12 UGUU motifs and those with fewer or equal to 12 in the 3′UTR region upstream to the polyadenylation sites (PAS). The *P*‐value was calculated using the Mann–Whitney *U* test (*p *< 0.001). Abbreviations: GO, Gene Ontology; IGV, Integrative Genomics Viewer; qPCR, quantitative polymerase chain reaction; sh, short hairpin; PAS, polyadenylation sites.

We performed photo‐activatable ribonucleoside cross‐linking and immunoprecipitation coupled with high‐throughput sequencing (PAR‐CLIP‐seq) in DAZ1‐overexpressing DAOY cells to identify the targeted transcripts of DAZ1. Overall, 2357 DAZ1‐bound mRNAs were identified in two biological replicates (Figure S4a, Table S6, Supporting Information). Further analysis indicated that DAZ1 bound mostly with the 3′‐UTR regions of the target mRNAs via the UGUU motif (**Figure** **4**a,b). To confirm that DAZ1 targeted mRNAs identified by PAR‐CLIP‐seq, RNA immunoprecipitation and RT‐qPCR assays were performed for three target mRNAs, namely, *DDX55* (DEAD‐Box Helicase 55), *RAD21* (RAD21 Cohesin Complex Component), and *SMC2* (Structural Maintenance of Chromosomes 2), which are involved in the biological processes of translation, proliferation, and cell cycle phase transition. The RIP‐qPCR results showed significant enrichment of these three mRNAs in anti‐DAZ immunoprecipitation of DAOY cells, indicating that DAZ protein can bind these mRNAs (Figure S4b, Supporting Information). Furthermore, Western blotting showed that the expression of these three proteins was decreased in *DAZ* knockdown cells than in control cells (Figure S4c, Supporting Information). However, RT‐qPCR showed that there was no significant difference in the RNA levels of these three targets, suggesting that the translation of the three target mRNAs was affected (Figure S4d, Supporting Information).

In parallel with the above analysis, we compared changes in the translation efficiency (TE) of DAZ‐targeted transcripts between *DAZ* knockdown cells and control cells by ribosome profiling coupled with high‐throughput sequencing (Ribo‐seq). In total, TE of 470 genes (Table S7, Supporting Information) was significantly downregulated (only genes with Ribo‐seq transcripts per million >1 and RNA transcripts per million >5, genes with a *p‐*value <0.05, and genes with a fold change >1.5 were considered as downregulated) in *DAZ* knockdown cell lines compared with control cells, and 103 of these transcripts overlapped with DAZ1‐bound mRNA (Figure 4c). Gene Ontology (GO) analysis of the downregulated DAZ1‐bound mRNAs revealed significant enrichment in the translation, ribonucleoprotein complex biogenesis, and chromosome segregation pathways (Figure 4d). Two DAZ1‐targeted mRNAs, *RAD21* and *SMC2*, which are involved in chromatid cohesion and sister chromatid cohesion from S‐phase DNA replication to their segregation during mitosis, may contribute to the progression of the cell cycle and proliferation of DAOY cells.^[^ ^36^, ^37^ ^]^ To confirm the results of Ribo‐seq (Figure 4e), we performed RT‐qPCR after polysome profiling (Figure 4f) and found that *RAD21* and *SMC2* transcript levels in the polysome fractions (the main portion of translationally active parts in polysome profiling) were significantly lower (*P‐*value <0.05) in *DAZ* knockdown cells than in control cells. However, the RNA‐seq results showed that mRNA expression levels of *RAD21* and *SMC2* were not significantly different between *DAZ* knockdown cells and control cells (Figure 4e). These findings demonstrated that the translation of DAZ target transcripts (*RAD21* and *SMC2*) was downregulated after knockdown of *DAZ*.

Integrative analysis of the DAZ1 PAR‐CLIP‐seq and Ribo‐seq data revealed a noticeable decrease in the TE of DAZ1 targets (796 transcripts) compared with nontargets (2089 transcripts) after knockdown of *DAZ* (*p *< 0.001, Mann–Whitney *U* test) (Figure 4g), suggesting that DAZ1 facilitates translation of its target mRNAs. We conducted an integrative analysis of PAR‐CLIP‐seq and Ribo‐seq to investigate whether the effect of DAZ‐enhanced translation is dependent on the number of binding sites in mRNAs. After re‐analyzing the UGUU occurrences in the 3′UTR region upstream to the polyadenylation sites (PAS) using the CLIP‐seq data (PAS, data from human cells were previously reported by Stroup et al.^[^ ^38^ ^]^), we sub‐grouped the DAZ's targets into two groups: Group 1 (motif occurrences≤12) and Group 2 (motif occurrences>12). It was shown that the translation efficiency was higher in the group with more than 12 motifs (*p *< 0.001, Figure 4h), indicating that the number of DAZ‐binding sites in mRNAs may be associated with DAZ‐enhanced translation. Also, we investigate whether the distance between DAZ binding sites and the poly‐A tail could play a significant role in controlling translation. We chose PAS as the starting point of the poly A tail and counted the median distance (number of nucleotides) between UGUU motif and PAS. Pearson correlation coefficient test showed a weak correlation between translation efficiency and the median distance (*r* = 0.24, *p *< 0.001, Figure S4e, Supporting Information), indicating the distance between DAZ binding sites and the poly A tail might only have a weak influence on the function of DAZ protein.

Notably, the transcript level of DAZ1‐targeted mRNAs was slightly higher in DAZ knockdown cells (Figure S4f, Supporting Information), suggesting that, unlike DAZL, DAZ is not required for the stabilization of its target mRNAs.^[^ ^18^ ^]^ These findings in the DAOY cells showed that DAZ was required for efficient translation of a group of target genes by binding to the 3′‐UTR of these targets, which were associated with proliferation and cell cycle phase transition.

### 2.5 DAZ Enhances Translation of Target mRNA via the UGUU Motif in Human Spermatogonia

**Figure 5:**
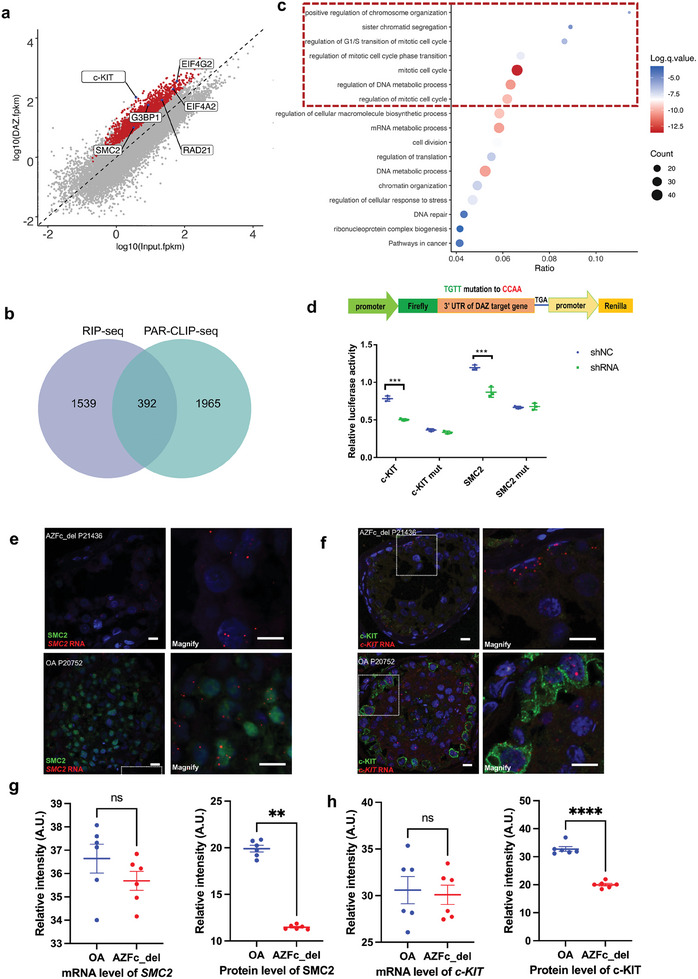
DAZ enhances the translation of its target mRNAs via the UGUU motif in the human testis. a) Scatter plot showing DAZ binding transcripts (*p *< 0.05, fold‐change >2) identified by RIP‐seq in human testicular tissue. b) Venn diagram showing the overlapped transcripts identified by RIP‐seq in human testis and by PAR‐CLIP‐seq in DAOY cells. c) GO analysis of DAZ targets detected by RIP‐seq overlapped with PAR‐CLIP‐seq. d) Schematic illustration showed the construction of dual luciferase reporter genes. Renilla luciferase was used as an internal control for normalizing firefly luciferase signals from different samples (shRNA vs shNC, Student's *t*‐test, two‐sided/unpaired). The data shown are the mean ± standard deviation (*n* = 3). ***P<0.001. NS, not statistically significant. e) RNA in situ hybridization combined with IF showed expression of SMC2 (panel e) in testicular tissue from patients with AZFc_del and OA controls. Scale bar, 10 µm. f) RNA ISH combined with IF showed expression of c‐KIT (panel f) in testicular tissue from patients with AZFc_del and OA controls. Scale bar, 10 µm. g) Quantification of mRNA and protein expression of SMC2 detected by RNA ISH combined with IF. Samples from OA controls and patients with AZFc_del (six fields examined for three biologically independent patients per group) were analyzed using the Student's *t*‐test (two‐sided/unpaired). The data shown are the mean ± standard deviation. ^**^ *p *< 0.01. ns, not statistically significant. h) Quantification of mRNA and protein expression of c‐KIT by RNA ISH combined with IF. Samples from OA controls and patients with AZFc_del (six fields examined for three biologically independent patients per group) were analyzed using the Student's *t*‐test (two‐sided/unpaired). The data shown are the mean ± standard deviation. ^****^ *p *< 0.0001. Abbreviations: ns, not statistically significant. AZFc, azoospermia factor c; AZFc_del, deletion of the AZFc region; GO, Gene Ontology; IF, immunofluorescence; ISH, in situ hybridization; OA, obstructive azoospermia; RIP, RNA co‐immunoprecipitation.

The target transcripts that DAZ bound in the human testis were identified using RIP‐seq. The RIP‐seq analysis identified 1941 DAZ target transcripts in the human testis in triplicate experiments (each containing three testes with OA) (Figure S5a, Table S8, Supporting Information; **Figure** **5**a). After combining the data from RIP‐seq of the human testis with those from PAR‐CLIP‐seq of DAOY cells, 392 overlapped DAZ target transcripts were identified (Figure 5b). Gene Ontology analysis showed that these genes were highly enriched in the “cell cycle” and “translation” pathways, consistent with the PAR‐CLIP‐seq results in DAOY cells (Figure 5c).

The stem cell growth factor receptor (*c‐KIT*) was also substantially enriched in the DAZ RIP‐seq of the human testis in addition to *SMC2* and *RAD21* identified in DAOY cells (Figure 5a). *c‐KIT* was highly transcribed in the human testis but poorly expressed in DAOY cells, so was not detected by PAR‐CLIP‐seq or Ribo‐seq of DAOY cells. Furthermore, *c‐KIT* and other DAZ target RNAs (including *SMC2* and *RAD21*) identified in DAOY cells were examined for DAZ binding in vivo via RIP‐RT‐qPCR in the human testis. All three mRNAs were immunoprecipitated by DAZ (Figure S5b, Supporting Information). An integrated analysis of the testicular RIP‐seq and scRNA‐seq data revealed that the expression of DAZ target mRNAs in samples from patients with AZFc_del was not significantly different from that in OA controls (Figure S5c, Supporting Information). Violin plots of the scRNA‐seq data showed that expression of *c‐KIT* and *SMC2* in spermatogonia was slightly higher in the AZFc_del group than in the control group (Figure S5d, Supporting Information).

Dual‐luciferase reporter assay was employed to determine whether DAZ can bind the 3′‐UTR of *c‐KIT* and *SMC2* in vitro. In cells transfected with plasmids carrying the 3′‐UTRs (200–300 bp containing the UGUU motif) of *c‐KIT* and *SMC2* inserted after the coding sequence of luciferase, the firefly luciferase signal was greater in control cells than in DAZ knockdown cells. After mutating UGUU (TGTT in the corresponding DNA sequence) to CCAA, there was no significant difference in the expression of firefly luciferase between the control cells and *DAZ* knockdown cells, indicating that DAZ protein can bind the 3′‐UTRs of *c‐KIT* and *SMC2* via the UGUU motif (Figure 5d). To determine whether translation of *c‐KIT* and *SMC2* was downregulated in patients with AZFc_del, expression of c‐KIT and SMC2 in the human testis were further investigated by RNA In Situ Hybridization Assay (RNA ISH) combined with IF using peptidylprolyl isomerase b (*PPIB*) as the positive control and the RNA polymerase II subunit A (*Polr2A*) as the negative control (Figure S5e, Supporting Information). We found that *c‐KIT* RNA and *SMC2* RNA were expressed in the same cells with DAZ expression in the human testis (Figure S5f, Supporting Information). The protein levels of c‐KIT and SMC2 were markedly lower in the AZFc_del samples than in the OA control samples, whereas the expression of mRNA detected by RNA‐ISH was slightly weaker but not significantly different (Figure 5e–h). Altogether, DAZ may play a role in the maintenance of c‐KIT‐positive spermatogonia by regulating the translation of target transcripts involving cell proliferation and cell cycle phase transition (e.g., *c‐KIT* and *SMC2*) in the human testis. Loss of *DAZ* accompanied by AZFc_del resulted in defective proliferation of spermatogonia and spermatogenic failure.

## 3 Discussion

**Figure 6:**
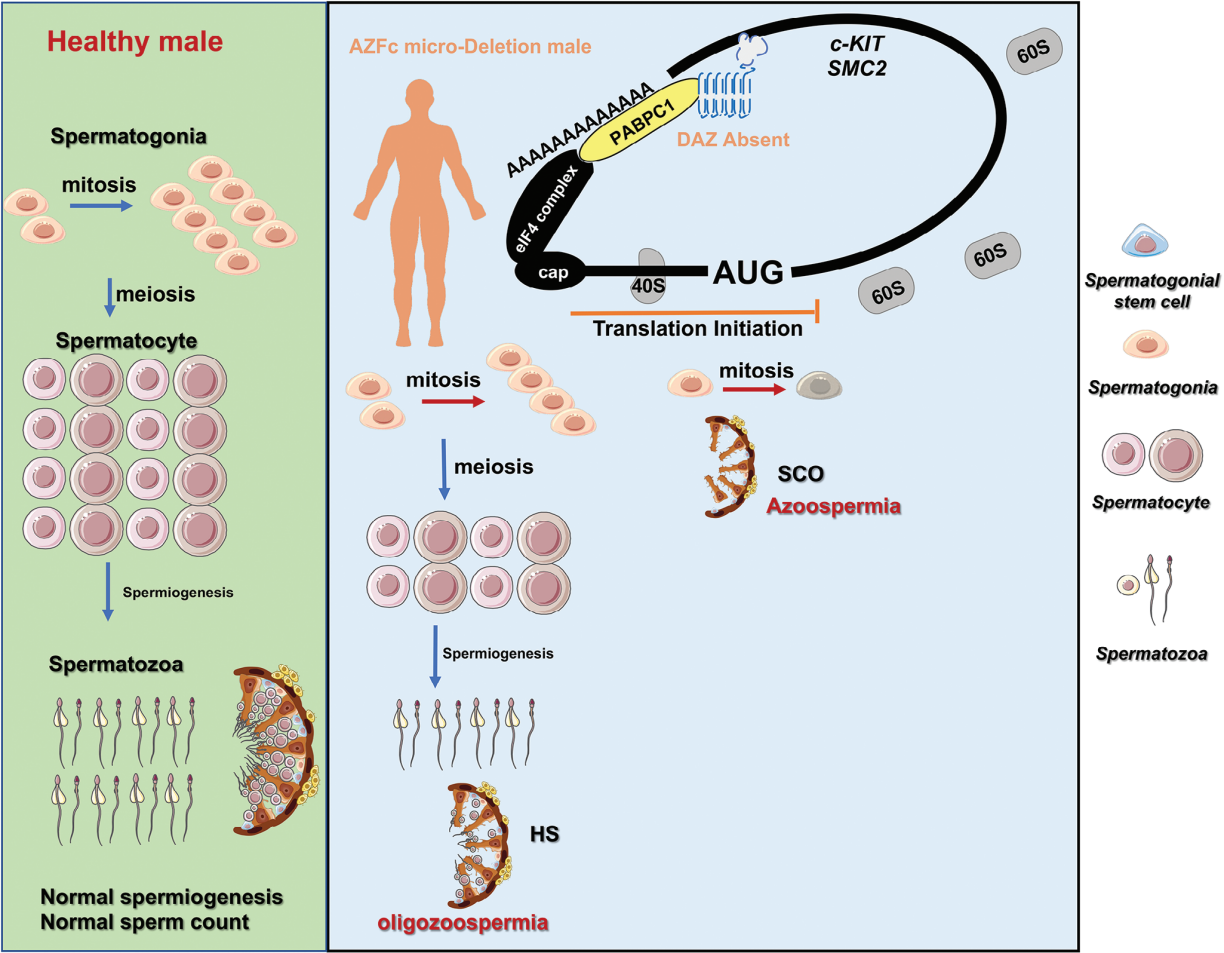
Loss of *DAZ* was associated with spermatogenic failure involving the defective proliferation of spermatogonia in patients with AZFc_del. Schematic representation of the molecular mechanisms of DAZ in a healthy man with normal spermatogenesis and the pathological mechanism in AZFc_del. Loss of *DAZ* decreases the efficient translation of mRNAs associated with proliferation and the cell cycle (such as *c‐KIT* and *SMC2*) and results in defective proliferation of c‐KIT‐positive spermatogonia, leading to azoospermia and severe oligozoospermia in patients with AZFc_del. Abbreviations: AZFc, azoospermia factor c; AZFc_del, deletion of the AZFc region; HS, hypospermia; MA, maturation arrest; SCO, Sertoli‐cell only.

Our comprehensive investigation of the molecular function of DAZ indicates that DAZ is required for the maintenance of c‐KIT‐positive spermatogonia. DAZ regulates the translation of target transcripts involved in cell proliferation and cell cycle phase transition (including *c‐KIT* and *SMC2*) in the human testis. Loss of *DAZ* accompanied by AZFc_del was associated with defective proliferation of spermatogonia and severe oligozoospermia or azoospermia (**Figure** **6**).

Our findings indicate that loss of *DAZ* decreased proliferation and cell cycle phase transition of c‐KIT‐positive spermatogonia, but had no obvious effect on the numbers of SSCs. c‐KIT is a specific marker of differentiating spermatogonia and a key regulator of cell proliferation in spermatogonia.^[^ ^39^, ^40^, ^41^ ^]^ In seminiferous tubules, deficiency of *c‐KIT* may lead to loss of spermatogonia. Therefore, there will be a decrease in the total number of spermatocytes and spermatids, and patients may exhibit oligozoospermia or azoospermia.

Our results demonstrate that DAZ enhances the translation of target mRNAs via the UGUU motif in human spermatogonia. We identified 2357 DAZ‐bound mRNAs by PAR‐CLIP‐seq in DAOY cells and 1941 DAZ target transcripts in the human testis by RIP‐seq. However, only 392 overlapping target transcripts were identified in both PAR‐CLIP‐seq and RIP‐seq. These results suggest that there are some differences between in vitro cell lines and human testicular tissue. For instance, *c‐KIT*, a marker of spermatogonia, which plays a key role in determining the fate of spermatogonial stem cells, was enriched by DAZ in RIP‐seq, but not expressed in DAOY cells. We selected the DAOY cell line that endogenously expresses DAZ for our study due to there is no suitable animal model to explore the function of DAZ. We found that DAZ affects cell proliferation by regulating of cell cycle in DAOY cells. Intriguingly, Single‐cell RNA‐seq profiling revealed that DAZ depletion is associated with deficient proliferation of spermatogonia in patients with AZFc_del. Furthermore, we identified that DAZ is involved in translational regulation in DAOY cells. In addition, through testicular in situ hybridization combined with immunofluorescent staining, we verified that the translation of DAZ target genes *SMC2* (enriched by PAR‐CLIP‐seq and RIP‐seq) and *c‐KIT* (only identified by RIP‐seq) was down‐regulated in the testis of DAZ deletion patients (with AZF_del). Therefore, we speculate that DAZ regulates the translation of spermatogonia via the same mechanism in human testis as in cells.

This study also suggests that *DAZ* gene which emerged during the evolution of primates and it may play a different role from that of *DAZL*. Previous studies have shown that DAZL regulates broad transcripts involving “progression of meiotic cell cycle” via the UGUU/GUU motif.^[^ ^16^, ^17^ ^]^ Given that the RRM domains of DAZ and DAZL lack binding specificity, both may bind the same motif to activate their target mRNAs in germ cells. However, there are several differences between human *DAZ* and *DAZL*. First, the findings of our present study demonstrate that DAZ is expressed mainly in c‐KIT‐positive spermatogonia whereas DAZL is abundantly expressed in spermatocytes, indicating that these two proteins play different roles in spermatogenesis. Second, targets of DAZ (such as *c‐KIT*, *RAD21*, and *SMC2*) are enriched in mitosis, unlike key genes involved in meiosis (such as *Rad51*, *Sycp1*, and *Sycp2*) or expansion and differentiation of progenitors of spermatogonia (such as Lin28, Sox3, Foxo1, and Sall4) targeted by mouse DAZL.^[^ ^16^, ^17^ ^]^ Collectively, both human DAZ and DAZL are indispensable for spermatogenesis, which may explain why human DAZL is unable to compensate for loss of DAZ in patients with AZFc_del.

We found that DAZ can bind PABPC1 via its DAZ repeat domain to regulate translation. We found that ribosome occupancy was significantly lower in *DAZ* knockdown cells than in control cells, which indicates that the overall cell translation level was significantly lower in cells with *DAZ* knockdown. Polysome profiling assay demonstrated that mRNA levels in the 40S and 60S fractions were higher in the DAZ knockdown cells than in the control cells, indicating that more immature ribosomes failed to assemble into 80S. Correspondingly, P/M (polysome/monosome) ratio decreased in the *DAZ* knockdown group, indicating a decrease in the number of mRNAs in the translation state. These findings demonstrated that the global translation level in cells is significantly reduced after the knockdown of *DAZ*. In addition, RIP‐qPCR assay of the various components of the ribosome showed that the polysome‐bound *SMC2* and *RAD21* were significantly reduced in the *DAZ* knockdown group than in the control group. This suggests that the translation of *SMC2* and *RAD21* was downregulated in the *DAZ* knockdown group. Our results also showed that DAZ can bind the CCT complex via the DAZ repeat domain in an RNA‐independent manner, indicating that DAZ could regulate the translation and protein folding of its target transcripts by interacting with PABPC1 and the CCT complex via the DAZ repeat domain. Previous studies reported that the CCT complex plays a role in protein folding.^[^ ^42^ ^]^ In the current study, we explored the molecular mechanisms of DAZ in translation regulation, and the exact biological function of DAZ in the regulation of the protein folding associated with CCT complex needs to be in‐depth of investigation in the future.

In summary, this study presents molecular evidence that loss of *DAZ* is associated with spermatogenic failure involving defective maintenance of spermatogonia in patients with AZFc_del. AZFc_del may decrease the proliferation of c‐KIT‐positive spermatogonia, leading to severe oligozoospermia and even azoospermia. Given that the absence of DAZ is a key factor in spermatogenic failure with AZFc_del, the development of protein replacement agents that target DAZ may be a promising avenue for the development of a therapeutic approach to treat spermatogenic failure in these patients.

## 4 Experimental Section

### Cell Culture

DAOY cells were cultured in a Minimum Essential Medium containing 10% fetal bovine serum (FBS). HEK 293T cells were cultured in Dulbecco's Modified Eagle Medium containing 10% FBS. All cell lines were cultured in a growth medium containing 1% penicillin–streptomycin (15‐140‐122; Thermo Fisher Scientific, Waltham, MA, USA) according to the manufacturer's instructions in an incubator with 5% CO_2_ at 37 °C.

### Validation of Reagents

DAOY cells (CTCC‐400‐0014; Meisen Cell Company, Hangzhou, China) were purchased from the Chinese Tissue Culture Collection (www.ctcc.online), for which the STR identification report can be seen in the Supporting Information. HEK 293T cells were kindly donated by Professor Chen Hao from the Department of Human Cell Biology and Genetics, School of Medicine, Southern University of Science and Technology. All antibodies used were purchased from commercial companies (Table S1, Supporting Information).

### Patients

The experiments performed in humans were approved by the ethics committee of Shanghai General Hospital (license number 2022SQ294). Written informed consent was obtained from the donors for the use of their clinical data and testicular tissues for research purposes. Patients diagnosed with obstructive azoospermia (OA) who had normal spermatogenesis were enrolled as OA controls. Three further AZFc microdeletion samples were obtained from tissues after microdissection testicular sperm extraction. Microdeletion of AZFc was detected by quantitative polymerase chain reaction (qPCR) analysis and next‐generation sequencing. The qPCR results showed that sY254 and sY255 (containing two DAZ gene clusters) were completely deleted in all AZFc_del samples. Next‐generation sequencing showed that b2–b4 (seq[GRCh37] del(Y)(q11.223q11.23) chrY:g.24872470_28297296del) were completely deleted in all AZFc_del samples. Donors with other abnormal genotypes related to spermatogenic disorders were excluded by karyotype analysis and whole‐exome sequencing.

### Hematoxylin‐Eosin Staining

The fresh testicular tissues were fixed in 4% paraformaldehyde for 12–24 h at 4 °C, followed by embedding in paraffin and sectioning. Before staining, the tissue sections were dewaxed in xylene, rehydrated using a gradient series of ethanol solutions, and washed in distilled water. Next, the sections were stained with hematoxylin‐eosin and then dehydrated using increasing concentrations (70–100%) of ethanol and xylene. Finally, the sections were sealed on coverslips using neutral resin.

### Immunofluorescence Staining

The paraffin sections were dewaxed and rehydrated using the method described previously. After rehydration, the sections were processed for antigen retrieval with sodium citrate buffer (pH 6.0) at 105 °C for 10 min. For cell processing, 2 × 10^4^ DAOY cells were seeded per 24‐well dish. After three washes in phosphate‐buffered saline (PBS), the cells were treated with 4% paraformaldehyde at room temperature for 30 min. The sections and slides were blocked with 5% normal donkey serum and incubated overnight with appropriate primary antibodies (Table S1, Supporting Information) at 4 °C. Next, the sections were washed three times in PBS and incubated further with the secondary antibodies for 1 h a room temperature. The nuclei were stained by incubation with DAPI for 10 min. Images were captured using an LSM 980 confocal laser scanning microscope (Carl Zeiss AG, Jena, Germany) with the best signal model and 63× objective lens magnification (1.4 effective NA, oil). Excitation wavelengths of 553 493, and 401 nm. respectively, and emission wavelengths of 568, 517, and 422 nm were used for the AF555, AF488, and AF405 channels.

### Isolation of Single Testicular Cells

Testicular tissues were enzymatically digested with 4 mg mL^−1^ collagenase type IV, 2.5 mg mL^−1^ hyaluronidase, and 1 mg mL^−1^ trypsin at 37 °C for 20 min. The cell suspension was then filtered through 40‐µm nylon mesh. Dead cells were removed by magnetic‐activated cell sorting using a Dead Cell Removal Kit (Miltenyi Biotec, Gaithersburg, MD, USA). The cells were then resuspended in PBS containing 0.05% bovine serum albumin before preparation of a 10× Genomics library.

### Protein Extraction and Western Blotting

DAOY cells were lysed in RIPA buffer (R0278; Sigma‐Aldrich, St Louis, MO, USA) containing 1% protease inhibitor cocktail (11836153001; Roche, Basel, Switzerland) for 30 min on ice. Following centrifugation at 12 000 g for 20 min at 4 °C, the protein concentration of the lysates was determined using a BCA kit (23227; Thermo Fisher Scientific). SDS‐PAGE (Sodium dodecyl sulfate‐polyacrylamide gel electrophoresis) was performed using 20 µg of lysate from each sample, and Western blotting was carried out as previously described.^[^ ^25^ ^]^ After conducting SDS‐PAGE on 10% gel at 95 V for 30 min followed by 120 V for 80 min to load and separate the protein samples, the samples were transferred to 0.45‐µm polyvinylidene fluoride membranes. The membranes were then blocked with 5% nonfat dried milk and incubated with the primary antibodies overnight at 4 °C. The next day, the membranes were washed three times with TBS containing 0.1% Tween and then incubated with horseradish peroxidase‐conjugated secondary antibodies for 1 h at room temperature. After three washes in TBS containing 0.1% Tween, the blots were detected by chemiluminescence (Chemi‐Doc XRS; Bio‐Rad Laboratories). The primary and secondary antibodies are listed in Table S1 (Supporting Information).

### Quantitative PCR and PCR of Genomic DNA

Total RNA was extracted using a FastPure Cell/Tissue Total RNA Isolation Kit (RC112‐01; Vazyme, Nanjing, China) according to the manufacturer's protocol. First, 1 µg of total RNA was used for reverse transcription (RT) following the protocol for the HiScript 1st Strand cDNA Synthesis Kit (R111‐01; Vazyme). Next, Quantitative PCR (qPCR) was performed using a QuantStudio 7 Flex real‐time PCR system (Applied Biosystems, Waltham, MA, USA) with ChamQ Universal SYBR qPCR Master Mix (Q711‐02; Vazyme). *GAPDH* or *ACTB* was used as the endogenous control and each reaction was performed in triplicate. All of the primers used for qPCR analysis are listed in Table S2 (Supporting Information).

For PCR of genomic DNA, we extracted genomic DNA from the peripheral blood of the patients with AZFc_del and OA controls using the TIANamp Genomic DNA Blood Kit (Tiangen, Beijing, China) following the manufacturer's instructions. The primers for *DAZ* are shown in Table S2 (Supporting Information). PCR was initiated at 94 °C for 5 min and performed under the following conditions: denaturation at 94 °C for 30 s, annealing at 60 °C for 45 s, and elongation at 72 °C for 45 s, repeated for 35 cycles, with a final extension step performed at 72 °C for 5 min. The PCR products were separated by electrophoresis on 2% agarose gel, visualized with ethidium bromide, and recorded as images (Chemi‐Doc XRS; Bio‐Rad Laboratories).

### Construction of Plasmids

The plasmids were constructed using a ClonExpress Ultra One‐Step Cloning Kit (C115‐01; Vazyme) according to the manufacturer's instructions. cDNA ORF Clone of DAZ1 (catalog number HG18434‐UT), DAZ2 (catalog number HG18434‐UT), DAZ3 (catalog number HG20372‐UT), and DAZ4 (catalog number HG20689‐UT) were purchased from Sino Biological (Beijing, China). The 3×HA‐pHAGE vectors were linearized using BamHI (R3136V) and XbaI (R0145V), both of which were sourced from New England Biolabs (Ipswich, MA, USA). The 3×FLAG‐pCDH vectors were linearized using XhoI (R0146V) and BamHI (R3136V) from New England Biolabs. The DAZ1‐FLAG‐3×HA‐phage, DAZ2‐FLAG‐3×HA‐phage, DAZ3‐FLAG‐3×HA‐phage, DAZ4‐FLAG‐3×HA‐phage, PABPC1‐3×HA‐phage, 1–400 PAPBC1‐3×HA‐phage, 401–619 PAPBC1‐3×HA‐phage, DAZ1‐FLAG‐pCDH, 1–496 DAZ1‐FLAG‐pCDH, and 497–744 DAZ1‐FLAG‐pCDH plasmids were generated using cDNA ORF Clone templates. Information on all of the In‐Fusion PCR primers used is provided in Table S2 (Supporting Information). The plasmids were extracted using a FastPure EndoFree Plasmid Mini Kit (DC203‐01; Vazyme) according to the manufacturer's instructions.

### Lentivirus Production and Cell Transfection

For the knockdown experiment, shRNA‐coding DNA fragments were synthesized and cloned into the pLKO.1 vector to create pLKO.1‐DAZ shRNA plasmids. The pLKO.1‐DAZ shRNA1, shRNA2, and shNC (negative control) plasmids were transfected into HEK 293T cells along with the packaging plasmids pMD2.G and pSPAX2 using Lipofectamine 3000 (Invitrogen, Waltham, MA, USA). At 48 h after transfection, the virus particles were harvested and filtered using a 0.45‐µm cell strainer. After reaching 70% confluence, DAOY cells were infected with the particles and 6 mg mL^−1^ polybrene (Sigma‐Aldrich). Twenty‐four hours later, the culture medium was changed to Dulbecco's Modified Eagle Medium with 10% FBS, and green fluorescence was observed under a fluorescence microscope after 72 h to ensure successful infection. For selection, 2 µg mL^−1^ puromycin (HY‐B1743A; MedChemExpress, Monmouth Junction, NJ, USA) was added to the culture medium. ≈5 days later, the successfully infected cells were cultured for further experiments.

### Cell Proliferation Assays

Viable shRNA1, shRNA2, and shNC DAOY cells (5 × 10^4^) were seeded in 24‐well plates and counted every 24 h for 3 days using an automatic cell counter (Countess 3; Invitrogen). The cells were stained with trypan blue (0.04%, 15250061; Gibco, Billings, MT, USA) before counting to exclude dead cells.

### Cell Cycle Analysis

For synchronization of the cell cultures, *DAZ* knockdown DAOY cells were seeded in 6‐well plates with growth medium with 10% FBS and incubated overnight. The cultures were then gently rinsed in PBS and switched to a serum‐free medium. After serum starvation for 12 h, the cells were passaged and released into the cell cycle by the addition of serum.^[^ ^26^ ^]^ Cell samples were harvested with trypsinization (25200072, 0.25%; Gibco) at indicated time points for fluorescence‐activated cell sorting (FACS) analysis. The staining procedures were performed using a Cycle Test Plus DNA Reagent Kit (340242; BD Biosciences, Franklin Lakes, NY, USA) according to the manufacturer's instructions. Cell cycle phase distributions were analyzed by flow cytometry (340242; BD Biosciences). The percentage of cells in each phase of the cell cycle was determined using Modfit software (Verity Platform Inc., Boston, MA, USA). The cell cycle state was identified according to an expression of cell cycle‐specific genes from the scRNA‐seq data (the details regarding bioinformatic analysis of the scRNA‐seq are shown in the Supporting Information). Cell cycle analysis was performed using the Seurat package (version 4.1.1) according to the operation manual (https://satijalab.org/seurat/articles/cell_cycle_vignette.html). The cycle phase‐specific genes are listed in Table S3 (Supporting Information).

### Annexin V‐PI Staining

Apoptosis was evaluated by flow cytometry using an Annexin V‐fluorescein isothiocyanate (FITC) apoptosis detection kit (A211; Vazyme) according to the manufacturer's instructions with Annexin V‐FITC staining with propidium iodide (PI). *DAZ* knockdown DAOY cells were washed in cold PBS and resuspended in a binding buffer for measurement of the annexin shift. The cells were then stained with 5 µL of FITC‐labelled Annexin and analyzed by flow cytometry. For double staining, 5 µL of PI (5 mg mL^−1^) was added 5 min before analysis by flow cytometry using a FACS Aria cell sorter (BD Biosciences). When measuring the proportion of apoptotic cells, the green signal is detectable in the FL1 channel (FITC); 10000 events per sample were gathered during the detection of red fluorescence of PI in the FL3 channel.

### TUNEL assay

An in situ cell death detection kit (C1086; Beyotime, Shanghai, China) was used to evaluate apoptosis in *DAZ* knockdown cells affected by shRNA according to the manufacturer's manual. The cells were treated with 20 mg mL^−1^ proteinase K (3115887001; Roche) for 15 min at room temperature and then incubated with dUTP labeling/terminal deoxynucleotidyl transferase enzyme buffer for 1 h in the dark. DAPI was used to stain the cell nuclei. Cells treated with PBS but without the enzyme buffer were used as the negative control. At least 500 cells were evaluated per sample under a Zeiss 980 fluorescence microscope (Carl Zeiss) with an excitation wavelength of 488 nm.

### In Situ Hybridization Combined with Immunofluorescence

To evaluate the expression levels of mRNA and protein in situ, in situ hybridization (ISH) and immunofluorescence (IF) staining were performed on testicular tissue slides according to the manufacturer's instructions (RNAscope 2.5 HD Reagent Kit‐RED, catalog number 322350 and Co‐Detection Ancillary Kit, catalog number 323180; ACDBio, Newark, CA, USA). Briefly, tissues were pretreated with hydrogen peroxide for 10 min and Protease Plus for 30 min at 25 °C followed by one wash in PBS. *c‐KIT* and *SMC2* probes (RNAscope probe Hs‐c‐KIT, catalog number 606 401 and Hs‐SMC2, catalog number 1160451‐C1) were added and hybridized in situ at 40 °C for 2 h. The subsequent amplification steps were performed according to the manufacturer's instructions. The slides were washed twice in PBST (1× PBS containing 0.25% Triton X‐100). Sections were blocked in PBST containing 5% normal donkey serum for 1 h at room temperature. Primary antibodies (c‐KIT, SMC2, and DAZ) were prepared in PBS containing 5% normal donkey serum and added to the slides. The slides were then incubated overnight at 4 °C, after which they were washed three times in PBST. Next, the slides were incubated with secondary antibodies and DAPI was prepared in PBST for 1 h at room temperature. After washing with PBST three times, the slides were cover‐slipped with Fluoromount‐G (Southern Biotech, Birmingham, AL, USA). Images were captured at 63× magnification (1.4 Oil) using an LSM 980 confocal laser scanning microscope (Carl Zeiss). ImageJ software was used to detect the unstandardized intensity of protein and RNA expression of *SMC2* and *c‐KIT*. Dual ISH‐IF (RNA ISH combined with IF) staining of the RNA polymerase II subunit A (*Polr2A*) with anti‐DAZ antibody from the OA controls was used as a negative control for detection of RNA intensity. Immunofluorescence analysis of goat antirabbit secondary antibody Alexa Fluor 488 (green) without the primary antibody in the human testis was performed as a negative control for the detection of protein intensity. Quantitative analysis of dual ISH‐IF staining using pictures from a single channel of fluorescent staining, and threshold conversion was carried out to measure the density. The standardized intensity was calculated by unstandardized intensity subtracted from the mean intensity of the corresponding negative controls. The difference between the two groups was compared using the *t*‐test (GraphPad Prism 9; GraphPad Software Inc., La Jolla, CA, USA).

### Co‐Immunoprecipitation

DAZ‐overexpressing DAOY cells were collected at 80% confluence from two 150‐mm dishes (1 × 10^7^) and lysed in 1 mL of lysis buffer (10 mm Tris–HCl pH 7.5, 1 mm EDTA, 1 mm EDTA, 150 mm NaCl, and 0.1% Triton X‐100) containing 1× protease inhibitor cocktail for 20 min on ice. The protein‐containing supernatants were extracted by centrifugation at 13 000 g for 15 min at 4 °C. Next, 10% of the lysate was kept to be used as the input control, and the remainder was mixed with 2 µg of antibody (DAZ, PABPC1, and CCT3, refer to Table S1, Supporting Information) and gently rotated at 4 °C for 2 h. The preimmunoprecipitated samples (500–1000 µg) were then incubated with either Flag‐beads (F2426; Sigma‐Aldrich) or Protein A/G magnetic beads (88803; Life Technologies, Carlsbad, CA, USA) under rotation at 4 °C for 2 h. The proteins associated with the Protein A/G magnetic beads were collected by placing the tubes into a magnetic stand and rinsing five times with immunoprecipitation washing buffer (10 mm Tris–HCl pH 7.5, 1 mm EDTA, 150 mm NaCl, and 0.1% Triton X‐100), after which they were subjected to Western blotting. The lysates were digested with RNase A (DC102; Vazyme) at room temperature for 15 min for the co‐immunoprecipitation assays.

### Sucrose Density Gradient Ultracentrifugation

shRNA1, sRNA2, and shNC DAOY and NCIN87 cells in two 150‐mm dishes (1 × 10^7^) were treated with 100 µg mL^−1^ cycloheximide (HY‐12320; MedChemExpress) at 37 °C for 10 min. The cells were then washed twice in ice‐cold PBS containing 100 µg mL^−1^ cycloheximide. Next, they were harvested in 500 µL of lysis buffer (20 mm Tris–HCl pH 7.5, 100 mm KCl, 5 mm MgCl_2_, 1% Triton X‐100, and 100 µg mL^−1^ cycloheximide) supplemented with 1× protease inhibitor cocktail (5892791001; Roche). The samples were then centrifuged at 13 000 g for 15 min at 4 °C. The supernatants were collected and absorbance was measured at 260 nm. The sucrose gradient (10–50%) was generated using a Gradient Master (BioComp Instruments, Fredericton, NB, Canada). Equal amounts from each group, as determined by the A260 value, were layered onto a 12‐mL sucrose gradient and centrifuged at 35 000 rpm in an SW‐41 Ti rotor (Beckman Coulter, Brea, CA, USA) for 3 h at 4 °C. After centrifugation, the fractions were collected, and absorbance at 260 nm was determined using a BioComp Piston Gradient Fractionator (BioComp Instruments). The fractions were then subjected to Western blotting and RT‐qPCR analysis.

### Luciferase Assay

The 3′‐untranslated region (3′‐UTR) of the DAZ targets (*c‐KIT* and *SMC2*) containing the wild‐type motif (UGUU) or mutant motif (CCAA) was inserted into a pmirGLO vector. The luciferase plasmids were transfected into HEK 293T cells. After transfection, the luciferase signal was detected using a Dual‐Glo Luciferase Assay System (Promega, Madison, WI, USA) and normalized to the wild‐type group.

### Statistics and Reproducibility

The data are shown as the mean ± standard deviation unless otherwise indicated. Statistical significance was determined using the Student's *t*‐test with GraphPad Prism 9 software (for the results in Figure 5d,g,h; Figures S4b, and S5b,d, Supporting Information; unpaired, two‐tailed), one‐way analysis of variance (for the results in Figures 2, 3, and 4f; Figures S2b,d, S3e, S4d, Supporting Information), or Pearson chi‐squared test (for the results in Figures 1f and 2f). The confidence interval was 95%. Statistical significance was determined using R software (version 4.0.3; R Foundation for Statistical Computing, Vienna, Austria) and the Wilcoxon test (for the results in Figure 4g; two‐sided), Mann–Whitney *U* test (for the results in Figure S4e, Supporting Information; Figure 4h; two‐sided), or the Student's *t*‐test (for the results in Figure S5c, Supporting Information; unpaired, two‐tailed). A *p*‐value of <0.05 was considered statistically significant. Statistical parameters are reported in the figures and their legends. Figures 2b,c,e,g, 3e,f,g, and 4f; Figures S2b,c,e, S3c,e, S4b,d, and S5b were based on results obtained twice independently.

More information about the bioinformatic analysis is provided in the Supplementary Methods, including single‐cell RNA‐seq of testicular cells, single‐cell RNA sequencing analysis, identification of differentially expressed genes, and Gene Ontology analysis, PAR‐CLIP‐seq, RIP‐seq, RIP‐qPCR, and analysis of the PAR‐CLIP‐seq, Ribo‐seq, RIP‐seq, and RNA‐seq data.

### Ethics Approval Statement

The experiments performed in humans were approved by the ethics committee of Shanghai General Hospital (license number 2022SQ294).

## Conflict of Interest

The authors declare no conflict of interest.

## Author Contributions

N.O, Y.W, S.X, and J.L contributed equally to this work. L.Z., C.H., and Y.C.C conceived and designed the study. O.N.J. performed most of the experiments and analyzed the data. O.N.J., Y.C.C., and C.H. analyzed the data and wrote the paper. C.H., L.Z., and Y.C.C read and revised the various versions of the manuscript. Z.Y.X, J.Z.Y., Z.J.P, and B.H.W. assisted with the immunofluorescence assays. Z.Y.Y. and X.M.G. assisted with flow cytometry. Z.C.W., S.X.Y., J.Y.X., W.D., and W.R.Q. assisted with the construction of plasmids. T.R.H., L.P., Z.E.L., C.W., and H.Y.H helped to collect the human testis tissue for clinical research. X.S., Z.L.Y., and L.J.Q. analyzed the single‐cell RNA‐seq data. W.Y.C. performed the bioinformatic analysis. All authors reviewed and approved the final version of the manuscript. L.Z., C.H., Y.C.C., and O.N.J. verified the original data.

## Supporting information

Supporting Information

Supplemental Table 1

Supplemental Table 2

Supplemental Table 3

Supplemental Table 4

Supplemental Table 5

Supplemental Table 6

Supplemental Table 7

Supplemental Table 8

## Data Availability

The data that support the findings of this study are available from the corresponding author upon reasonable request.
