# Supplementary material for: Primate‐Specific DAZ Regulates Translation of Cell Proliferation‐Related mRNAs and is Essential for Maintenance of Spermatogonia
Source: Adv Sci (Weinh). 2024 May 23;11(29):2400692. doi: 10.1002/advs.202400692 (PMC11304246; doi:10.1002/advs.202400692)
Supplement: Supplementary file 1 — Supporting Information [file ADVS-11-2400692-s001.docx]

Supplementary materials for

**Primate-specific DAZ regulates translation of cell proliferation-related mRNAs and is essential for maintenance of spermatogonia**

**Ou *et al*.**

**Supplementary information**

**Tables and original data of Western blots**

Supplementary Table 1 Antibodies

Supplementary Table 2 Primers

Supplementary Table 3 A list of markers used to assign cell identity for scRNA-seq

Supplementary Table 4 Clinical parameters of Yq AZFc patients and OA controls enrolled

Supplementary Table 5 IP-DAZ1-MS in DAOY cell line

Supplementary Table 6 PAR-CLIP-seq gene list

Supplementary Table 7 Ribo-seq gene list (protein coding)

Supplementary Table 8 RIP-seq enriched protein coding genes

Supplementary Western blots

Verification of DAOY cells

**Supplementary Figure legends**

**Supplementary Methods**

**Supplementary Figure legends**


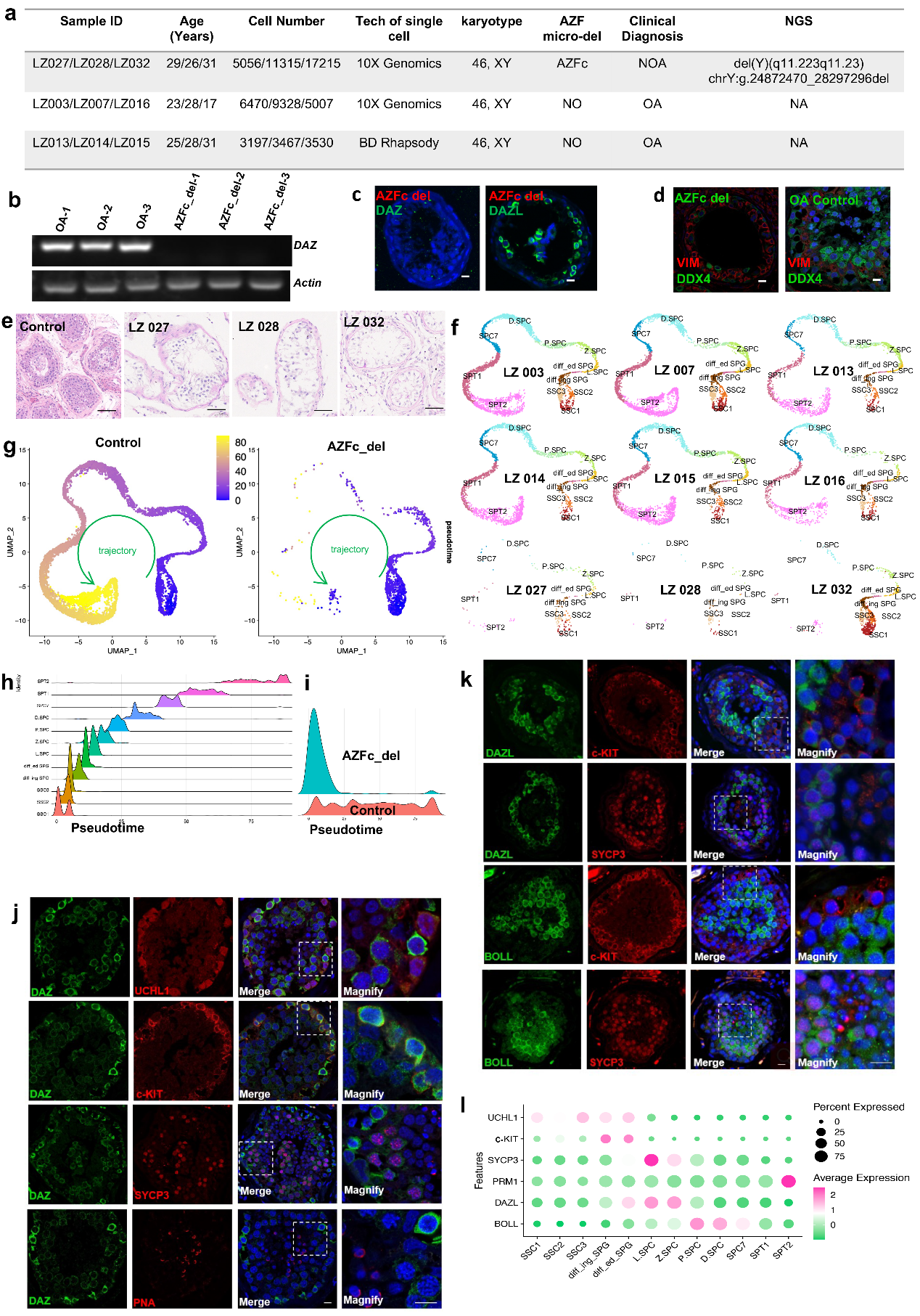


**Figure S1** **Clinical information of enrolled samples** **and expression of DAZ family genes in human testis**

a, Clinical information of enrolled samples in this study.

b, PCR showed identification of *DAZ* in all gDNA of AZFc_del samples and OA controls.

c, IF showed DAZ and DAZL expression in human testes from patients with AZFc_del. Scale bar=10 μm.

d, IF showed expression of DDX4 (marker of germ cells) and VIM (marker of Sertoli cells) in human testis from patients with AZFc_del and OA control. Scale bar=10 μm.

e, Periodic acid–Schiff (PAS) staining of each sample enrolled in this study illustrated the normal spermatogenesis in OA controls and spermatogenic disorder in the testis of patients with AZFc_del (LZ 027, LZ 028, LZ032).

f, UMAP plots of all germ cells from 6 OA samples (LZ 003, LZ007, LZ013, LZ014, LZ015, LZ016) and 3 cases of AZFc_del (LZ027, LZ028, LZ032), respectively.

g, UMAP representation of stages in spermatogenesis colored by pseudotime. The plot illustrates the trajectory of spermatogenesis progression, with colors indicating the inferred pseudotime along the trajectory. Early developmental stages are represented by cooler colors (blue), while later stages are depicted with warmer colors (yellow). Each point represents a single cell, and the distance between points reflects their similarity in gene expression patterns.

h, Ridgeline plot showed different pseudotime germ cell states.

i, Ridgeline plot showed different pseudotime germ cell states in human testis from patients with AZFc_del and OA controls, respectively.

j, k, IF (Immunofluorescence) staining for UCHL1 (spermatogonial stem cell marker), c-KIT (spermatogonia marker), SYCP3 (spermatocyte marker) and PNA (spermatid marker) with DAZ family (DAZ, DAZL, and BOLL) in testicular paraffin sections of OA controls. Scale bar=10 μm.

l, Bubble chart showed the expression of *DAZ* family genes and different germ cell markers in human germ cells of different stage according to scRNA-seq of OA samples.

Abbreviations: AZFc, azoospermia factor c; AZFc_del, deletion of the AZFc region; OA, obstructive azoospermia.


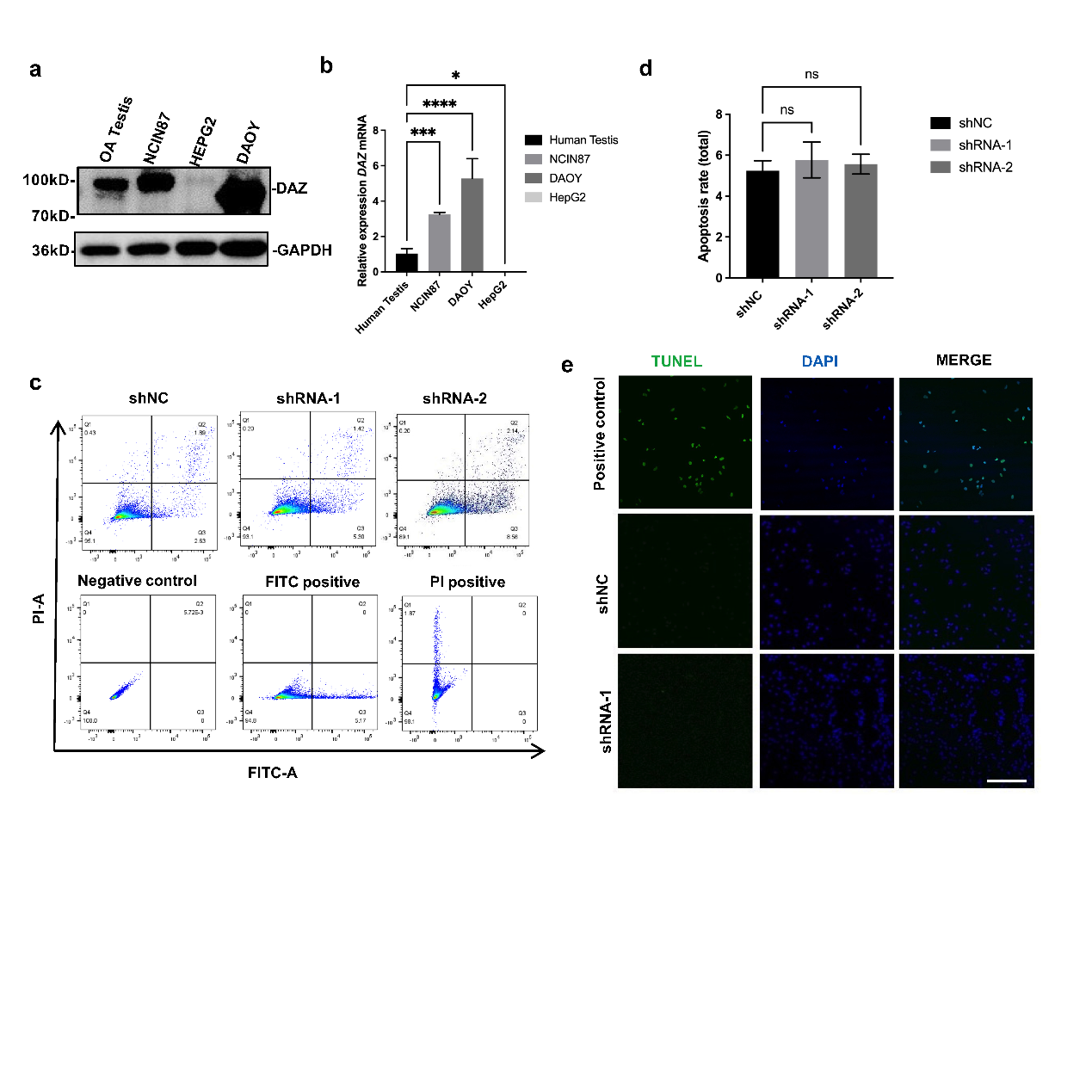


**Figure S2** **DAZ was highly expressed in DAOY cell line and deficiency of *DAZ* could not induce apoptosis**

a, Western blotting showed the expression of DAZ in different cell lines (NCIN87, HepG2, and DAOY cells) and OA testis.

b, qPCR results showed the expression of *DAZ* in different cell lines. The mRNA level of *DAZ* in DAOY, NCIN87 and HepG2 (negative control) comparing with human testis (positive control) using one-way ANOVA test, data shown are the means ± standard deviation (SD), n=3. ****indicated that *p* < 0.0001; ***indicated that *p* < 0.001; *indicated that *p* < 0.05.

c, Flow cytometry of Annexin V and PI stain showed the apoptosis of DAOY cells after *DAZ* knock-down. NC, negative control; FITC, Fluorescein 5-isothiocyanate; PI, Propidium Iodide.

d, Bar plot showed the proportion of apoptosis rate of DAOY cells after *DAZ* knockdown (shRNA1 and shRNA2 comparing with shNC using analysis of one-way ANOVA test, n=3). ns indicated no significance.

e, TUNEL stain showed the apoptosis of DAOY cells after *DAZ* knock-down. The treatment of DNase 1 (10U/ml) in shNC samples at room temperature for 10 minutes was used as the positive control. Green indicated the TUNEL positive cells. Scale bar=50 μm.


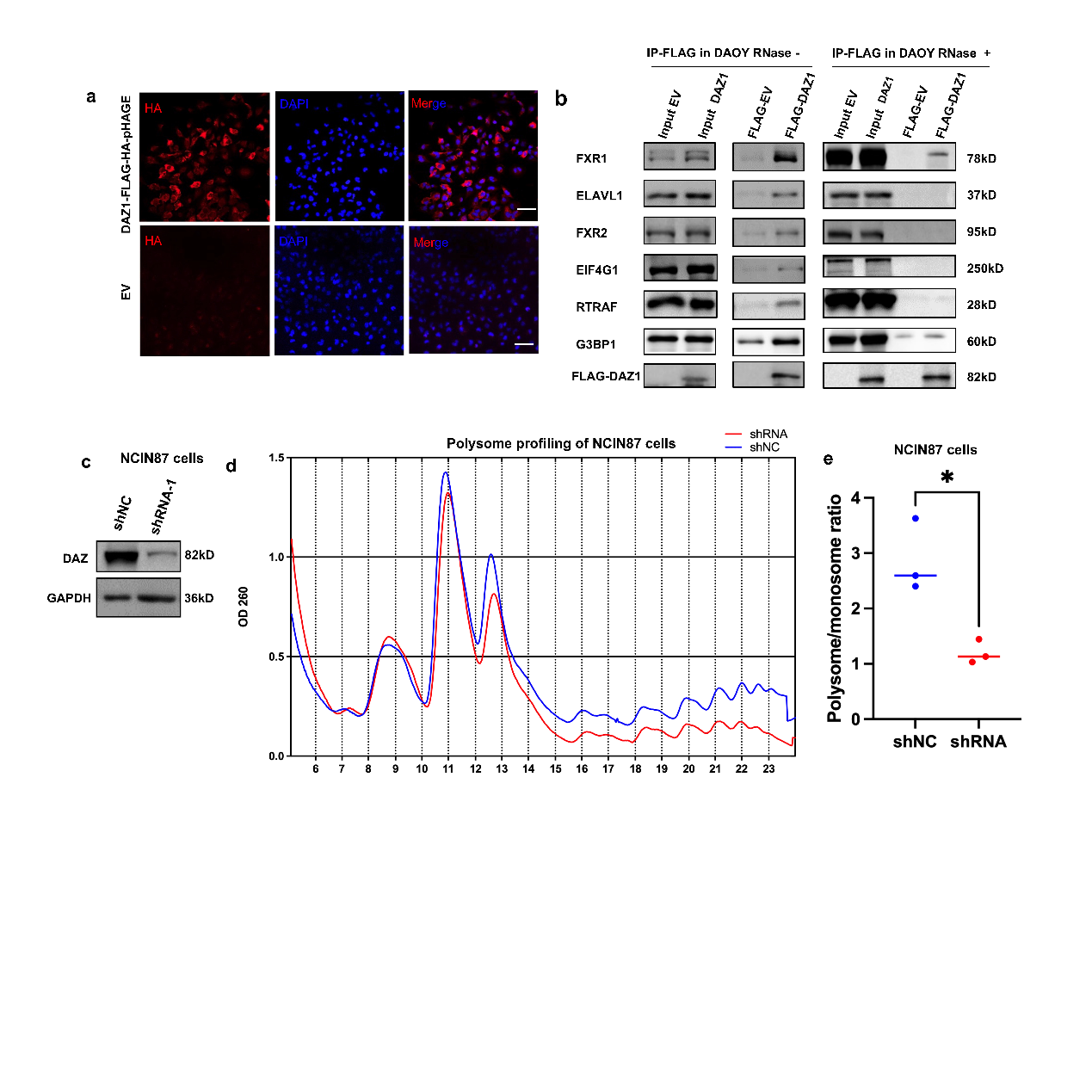


**Figure S3** **Construction of DAZ overexpression stable cell line and the verification of the interaction between DAZ and proteins identified by IP-MS**

a, Immunocytochemistry showed the expression of DAZ in DAOY cells transfected with plasmids (pHAGE) containing coding sequence of *DAZ1* and empty vector. Scale bar=10 μm.

b, co-IP showed interaction (with or without RNase A) between DAZ1 and proteins, which were assumed as translational regulators identified in mass spectrum, including FXR1, ELAVL1, FXR2, EIF4G1, RTRAF, and G3BP1. FXR1 (FMR1 Autosomal Homolog 1), FXR2(FMR1 Autosomal Homolog 2), ELAVL1(ELAV Like RNA Binding Protein 1), EIF4G1(Eukaryotic Translation Initiation Factor 4 Gamma 1), RTRAF (RNA Transcription, Translation and Transport Factor), and G3BP1(G3BP Stress Granule Assembly Factor 1).

c, Western blotting showed the expression of DAZ in NCIN87 cells after downregulation.

d, Polysome profiling showed the change of global translation after *DAZ* knockdown in NCIN87 cells.

e, Scatter plot showed the polysome/monosome ratio of NCIN87 cells after *DAZ* knockdown (shRNA vs shNC using one-way ANOVA). The data are shown as the mean ± standard deviation (n=3). **P*<0.05.

Abbreviations: NC, negative control; sh, short hairpin; ANOVA, analysis of variance.


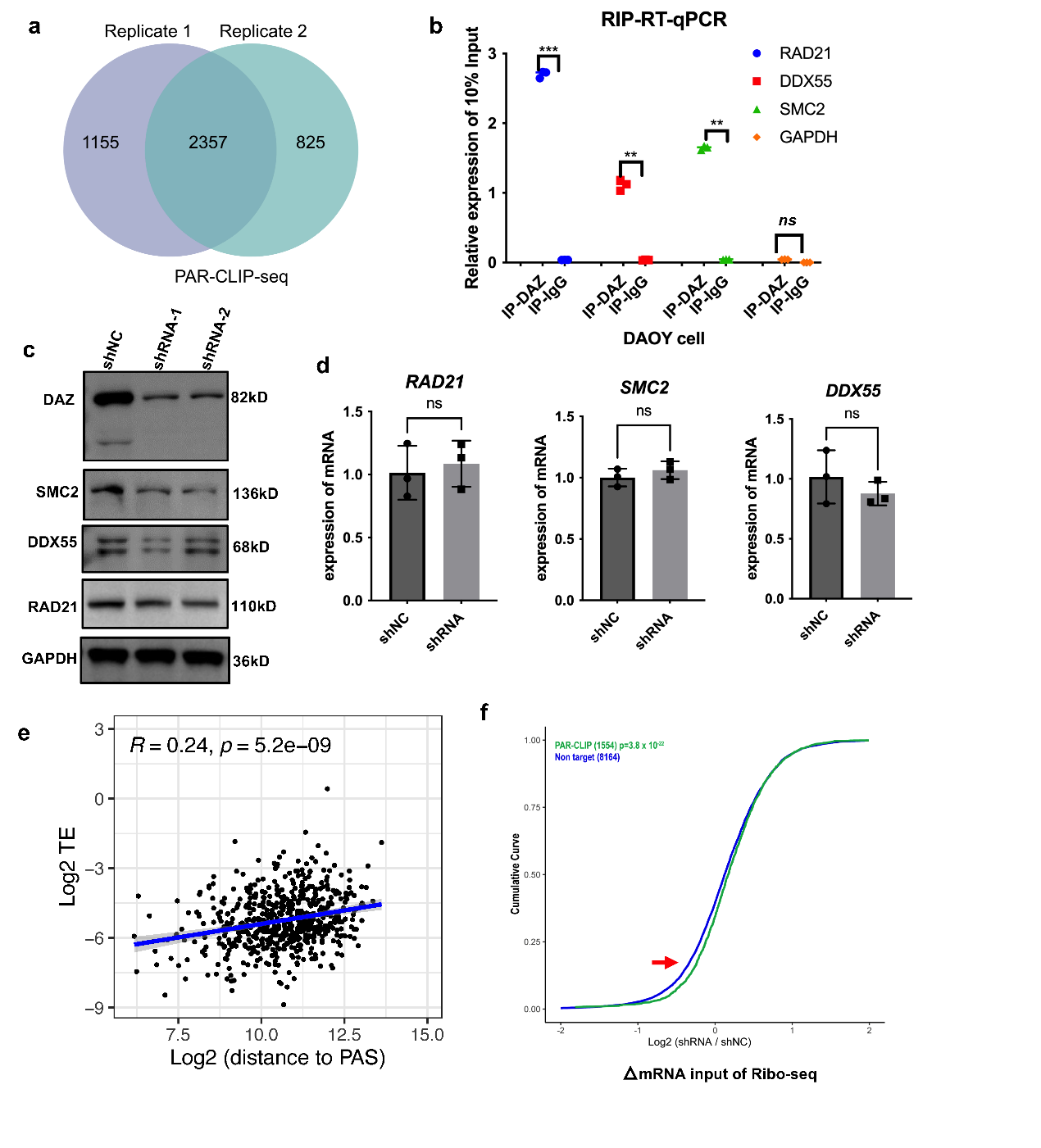


**Figure S4 DAZ promotes translation of target transcripts but not required for the stability of target mRNAs**

a, Venn diagram showed the overlap of DAZ PAR-CLIP-seq target genes between two biological replicates.

b, RIP/qPCR analyses showed the binding of DAZ and potential targets in wild-type DAOY cells. (IP-DAZ comparing with IP-IgG using Student’s t-test, two-sided/unpaired, data shown are the means ± standard deviation (SD), n=3). ***indicated that *p* < 0.001; **indicated that *p* < 0.01. ns, no significance.

c, Western blotting of DAZ target expression in DAZ knockdown and control cells, including SMC2, DDX55, and RAD21. DDX55, (DEAD-Box Helicase 55), RAD21, (RAD21 Cohesin Complex Component), and SMC2 (Structural Maintenance of Chromosomes 2).

d, RT-qPCR assays showed the expression of DAZ-Target transcripts (*SMC2*, *DDX55*, and *RAD21*) in DAZ knockdown DAOY cells. (shRNA vs shNC using one-way ANOVA). The data are shown as the mean ± standard deviation (n=3). ns, not statistically significant.

e, Scatter plot distribution of translation efficiency and median distance between UGUU motif and PAS in the 3′UTR in DAZ-target transcripts. The *P*-value was calculated using the Pearson correlation coefficient test, r = 0.24, *P*<0.001.

f, After the integration of RNA-seq with PAR-CLIP-seq, protein-coding genes with a sufficient expression level (RNA TPM >5) were divided into groups RAR-CLIP-targeted gene and non-PAR-CLIP-targeted gene groups. The empirical cumulative distribution function was performed based on the shRNA/shNC value. *P* values were calculated using a two-sided Mann-Whitney test. The red arrow between the green and blue lines around x=-0.5 to highlight the increase in mRNA level in DAZ target vs non-target mRNAs.

Abbreviations: NC, negative control; sh, short hairpin; ANOVA, analysis of variance; PAS, polyadenylation sites.


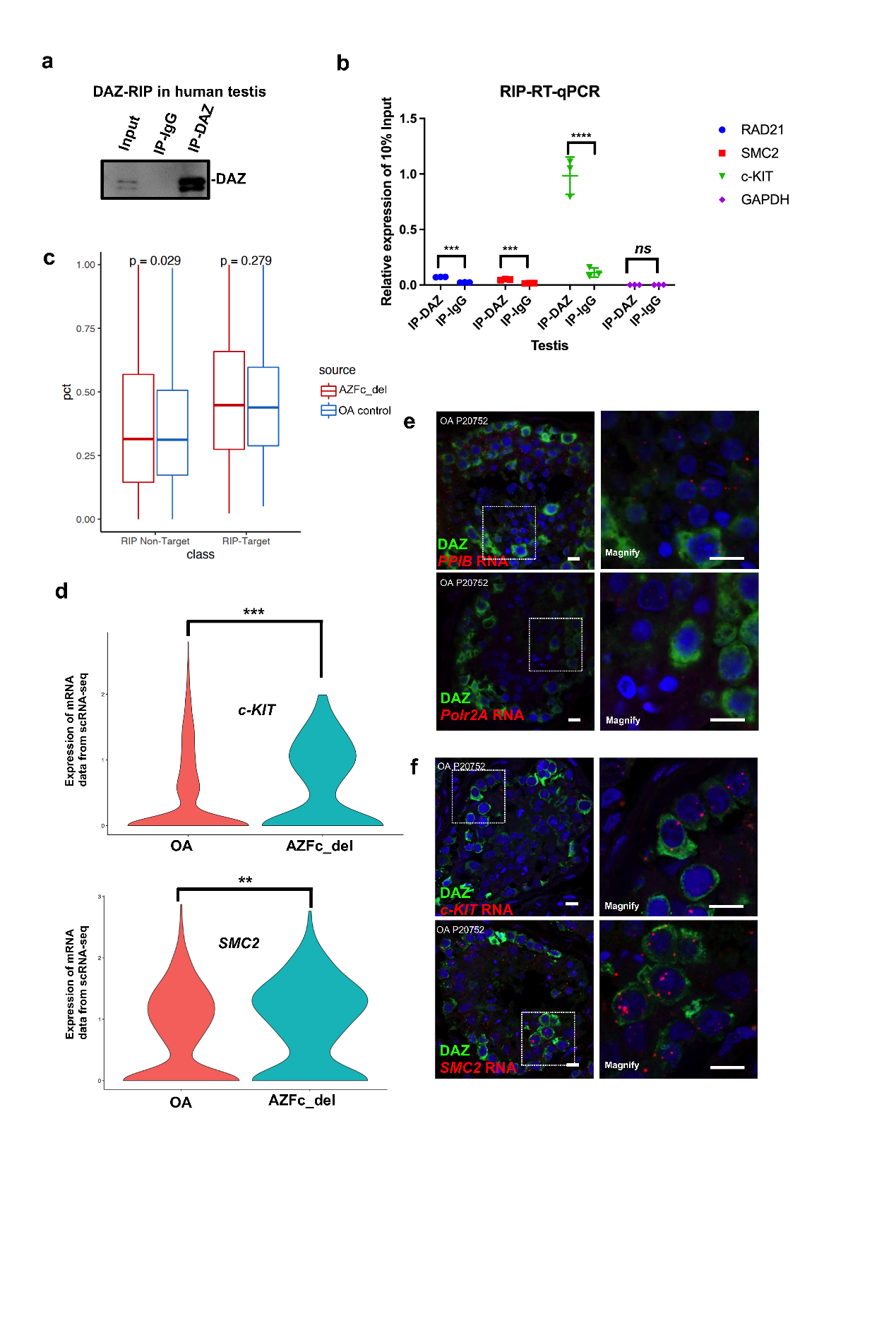


**Figure S5 Translation were down regulated in testis from patients with AZFc_del without affecting the stability of target mRNAs**

a, Western blotting showed the efficiency of IP-DAZ in five human testes with normal spermatogenesis.

b, RIP combined with RT-qPCR verified the enrichment of DAZ target transcripts (*RAD21*, *SMC2*, and *c-KIT*) in human testis. (IP-DAZ comparing with IP-IgG using Student’s t-test, two-sided/unpaired, data shown are the means ± standard deviation (SD), n=3). ****indicated that *P* < 0.0001; ***indicated that *P* < 0.001. NS, not statistically significant. *RAD21* (RAD21 Cohesin Complex Component), *SMC2* (Structural Maintenance of Chromosomes 2), and *c-KIT* (KIT Proto-Oncogene).

c, Integrative analysis of the testicular scRNA-seq and RIP -seq, transcripts from AZFc_del and control samples were divided into RIP-targeted genes and non-RIP-targeted genes. The *P* value between groups was calculated in Student’s t-test, two-sided/unpaired.

d, Violin plots showed the expression of *c-KIT* and *SMC2* in spermatogonia between the control group and AZFc_del group in scRNA-seq data. Statistical analysis between patients with OA controls and AZFc_del was conducted by two-tailed, unpaired Student’s t-test. The expression of *c-KIT* (p＜0.001) and *SMC2* (p＜0.01) was slightly higher in patients AZFc_del than OA controls. ***indicated that *P* < 0.001, **indicated that *P* < 0.01.

e, f, *c-KIT* RNA and *SMC2* RNA were expressed in the same cells with DAZ expression in the human testis detected by RNA ISH (in situ hybridization) combined with IF. PPIB and Polr2A are used as positive control and negative control to detect mRNA by RNA probe, separately. IF, immunofluorescence; *PPIB*, Peptidylprolyl Isomerase B; *Polr2A*, RNA Polymerase II Subunit A. Scale bar=10 μm.

**Supplementary methods**

**Single-cell RNA-seq of testicular cells**

The library was prepared as previously reported [1]. Briefly, cell suspensions were loaded on a Chromium Single Cell Controller instrument (10× Genomics, Pleasanton, CA, USA) to generate single-cell gel beads in emulsion. Single-cell RNA-seq libraries were then prepared using the Chromium Single Cell 3’ Library & Gel Bead Kit (P/N 120237, 10× Genomics) according to the manufacturer’s instructions. Sequencing libraries were generated from amplified complementary DNA (cDNA) using a 10× chromium kit, which included reagents for fragmentation, sequencing adaptor ligation, and sample index PCR. The final libraries were sequenced on an HiSeq ×10 platform (Novaseq 6000; Illumina, San Diego, CA, USA).

**Single-cell RNA sequencing analysis**

The pipeline includes mapping, sample quality control, and integration. Cell Ranger software (version 2.2.0) provided by 10 × Genomics was used to demultiplex cellular barcodes, map reads to the genome and transcriptome using the STAR aligner, and down sample reads as required to generate normalised aggregate data across samples, producing a matrix of gene counts versus cells. After mapping, the filtered count matrices for each sample were tagged with a special library batch ID, then a Seurat object was created using the Seurat package in R. Cells were further filtered according to the following threshold parameters: total number of expressed genes, 500–6000; total UMI count, between − ∞ and 35,000; and proportion of mitochondrial genes expressed, < 20%. Normalisation was performed according to the package manual (https://satijalab.org/seurat/v3.1/pbmc3k_tutorial.html). Samples (LZ003/LZ007/LZ016/LZ027/LZ028/LZ032) captured with 10× Genomics were considered as six independent batches, while samples (LZ013 /LZ014 /LZ015) were captured in the same batch using the Rhapsody system (BD Biosciences). The cell number captured by each sample were presented in **Supplementary Fig. S1a**. Batch correction was performed using the Integrate Data function in the Seurat package. The merged Seurat objects were scaled and analysed by principal component analysis (PCA). Then the ﬁrst 20 principal components (PCs) were used to construct a KNN (K-Nearest Neighbors Algorithm) graph and reﬁne the edge weights between any two cells. Based on all these cells’ local neighborhoods, the Find Clusters function with the resolution parameter set as 0.4 was used to cluster the cells. The ﬁrst 20 PCs were also used to perform non-linear dimensional reduction by UMAP, the detailed markers used for cell identity for scRNA-seq can be found in **Supplementary Table 2**, and the dimension reduction plots were given as output (**Fig. 1b, 1d and Fig. S1f**). For further analysis, we isolated germ cells and repeated these steps (**Fig. 1c, 1e**). Trajectory analysis of germ cells were conducted with Monocle3 (**Fig. S1g-S1i**).

Cell cycle state was identified according to the expression of cell cycle speciﬁc genes from scRNA-seq data. Cell cycle analysis were constructed with the Seurat package (version 4.1.1) according to the operation manual (<https://satijalab.org/seurat/articles/cell_cycle_vignette.html>). The cycle phase-speciﬁc genes were listed in **Supplementary Table 3**.

**Identiﬁcation of differentially expressed genes and Gene Ontology analysis**

The Seurat FindMarkers function (test.use = wilcox) is based on the normalized UMI count to identify differentially expressed genes (DEGs) between OA and AZFc_del samples. Unless otherwise noted, only the genes that were detected in at least 10% of the cells were tested, and the average log2 (fold change) threshold was set as 1 in the analysis. And gene ontology (GO) analysis was conducted on the DEGs using Metascape (https://metascape.org/gp/index.html).

**Photo-activatable ribonucleoside cross-linking and immunoprecipitation** **coupled with high-throughput sequencing**

Photo-activatable ribonucleoside cross-linking and immunoprecipitation coupled with high-throughput sequencing (PAR-CLIP-seq) was performed as previously described [2]. Briefly, Flag-3×HA-tagged DAOY cells (1×10^7^) stably expressing DAZ1 at 60% confluency (in a 150-mm dish) were incubated with 100 μM 4-thiouridine (13957-31-8; Sigma-Aldrich) for 14–16 h before crosslinking. Cells were washed with 10 ml of ice-cold PBS per plate, after which the plates were placed on a tray with ice and irradiated uncovered with 0.15 J/cm^2^ of 365 nm ultraviolet light in a Stratalinker 2400 crosslinker (Stratagene California, La Jolla, CA, USA). The cells were scraped off with cell scraper in 5 ml of ice-cold PBS per plate, and the cell pellets were collected by centrifugation at 500 g for 5 min at 4°C. Three volumes of 1× NP40 lysis buffer (50 mM HEPES, pH 7.5, 150 mM KCl, 2 mM EDTA, 0.5% [v/v] NP40, 0.5 mM DTT, 1× protease inhibitor cocktail) were added in one volume of cell pellet and incubated on ice for 10 min. The cell lysate was cleared by centrifugation at 13,000 g for 15 min at 4°C. Next, 1 U/μl RNase T1 (AM2283; Invitrogen) was added to the cell lysate, which was then incubated in a water bath for 15 min at 22°C. Then, the reaction was cooled for 5 min on ice before proceeding. The RNase T1-treated cell lysate was incubated with 25 μl of anti-HA magnetic beads (88836; Pierce Biotechnology, Waltham, MA, USA) on a rotating wheel for 3 h at 4°C. The beads were washed thrice in 1 ml of immunoprecipitation wash buffer (10 mM Tris–HCl pH 7.5, 1 mM EDTA, 150 mM NaCl, and 0·1% Triton X-100). The beads were treated with 1-10 U/μl RNase T1 in 50 μl of cell lysis buffer for 15 min at 22°C. The cell lysate was then cooled on ice for 5 min, after which the beads were washed thrice in 1 ml of high-salt wash buffer. The RNA fragments in the DAZ1-RNA complex were extracted via ethanol precipitation after digestion of the beads using proteinase K. The purified RNA pellet was dissolved in 12 µl of RNase-free water, 6 µl of which were subjected to small RNA library preparation with NEBNext^®^ Multiplex Small RNA Library Prep Set for Illumina^®^ (E7300S; New England Biolabs).

**RIP-seq and** **RIP-qPCR**

The RNA immunoprecipitation experiment was conducted according to manufacturer’s instructions with minor modifications. Briefly, 100 mg of human testis or (1×10^7^) DAOY cells in a 150-mm dish were subjected to RIP-seq and RIP-qPCR. For testicular tissue, the testis was ground with liquid nitrogen. Ground testis or cell pellets were lysed in RIP buffer (25 mM Tris, pH 7.4, 150 mM KCl, 2 mM EDTA, 0·5% [v/v] NP40, 0·5 mM DTT, 1× protease inhibitor cocktail). The mRNAs were then pulled down by antibody following the immunoprecipitation protocol. The co-precipitated RNA was recovered from the beads using a RNeasy mini kit (Qiagen Inc., Valencia, CA, USA). Both the input and co-immunoprecipitated RNA were recovered using an RNA Clean & Concentrator-5 kit (R1013; Zymo Research, Irvine, CA, USA) and subjected to RNA-seq or RT-qPCR analyses. The primers are shown in **Supplementary Table 2**.

**Ribosome profiling coupled with high-throughput sequencing**

Ribosome profiling coupled with high-throughput sequencing (Ribo-seq) was performed as previously described [3] with minor modifications. Cells at 70% confluency were treated with 100 μg/ml cycloheximide (C1988; Sigma-Aldrich) for 8 min at 37°C to prevent translation before lysis in ice-cold lysis buffer (20 mM Tris–HCl pH 7.4, 150 mM NaCl, 5 mM MgCl_2_, 1 mM DTT, 100 μg/ml cycloheximide, 1% vol/vol Triton X-100) for 10 min on ice. The lysates were collected by centrifugation at 20,000 g for 10 min at 4°C, and 10% of each cell lysate was kept as the input control and the remainder were prepared for RNA-seq after depletion of rRNA. The RNA concentration in the cell lysates was determined using a Qubit RNA HS assay kit (Q32852; Thermo Fisher Scientific) according to the manufacturer’s instructions. Polysome buffer (20 mM Tris–HCl pH 7.4, 150 mM NaCl, 5 mM MgCl_2_, 1 mM DTT, and 100 μg/ml cycloheximide) was then added to the lysates (30 μg of total RNA) to a total volume of 200 μl along with 1.5 μl of RNase I (N6910K; Epicentre, Durban, South Africa). The lysates were then incubated with gentle rotation for 45 min at room temperature. Nuclease digestion was quenched by addition of 10 μl of SUPERaseIn RNase inhibitor (AM2694, Invitrogen). The lysates were then loaded onto a 0.9-ml sucrose cushion (1 M sucrose and 20 U/ml SUPERaseIn RNase inhibitor in polysome buffer, pH 7.5) in a polypropylene tube (344625; Beckman Coulter). The ribosomes were pelleted by centrifugation at 220,000 g in a TLA-120.2 rotor at 4°C for 2 h and RNA samples were recovered from the ribosomal pellet using QIAzol (79306; Qiagen). The RNA samples were separated on 15% polyacrylamide TBE–urea gel (EC68852BOX; Invitrogen), and the gel band between 17 and 34 nucleotides was excised and collected. The footprint RNA was recovered from the gel by addition of 400 μl of RNA gel extraction buffer (300 nM NaOAc pH 5.5, 1 mM EDTA, 0.25% vol/vol sodium dodecyl sulphate and 0.1 U/ml SUPERase In RNase inhibitor, pH 7.5). After being frozen on dry ice for 30 min, the samples were allowed to thaw overnight at 25°C with gentle rotation, and the footprint fragments were purified by precipitation with isopropanol. The libraries were constructed using a NEBNext small RNA prep kit (E7330S; New England BioLabs) after depletion of rRNA and sequenced on an Illumina Hiseq 2500 system with a single-end read length of 51 base pairs.

**Analysis of PAR-CLIP-seq, Ribo-seq, RIP-seq, and RNA-seq data**

For pre-processing of sequencing data**,** the PAR-CLIP-seq, Ribo-seq, RIP-seq, and RNA-seq data were pre-processed as described previously [4]. In brief, adapters and low-quality bases were trimmed with TrimGalore (version 0.6.7). The clean data were first mapped to rRNA and tRNA sequences from the Ensembl database (release 104) and repetitive sequences from the Repbase (RepBase27.04) by Bowtie (version 1.3.1) [5], and the remaining unaligned reads were demultiplexed for further analysis.

For analysis of PAR-CLIP-seq data**,** processed reads were aligned to the reference genome (GRCh38) by STAR (version 2.7.10a) [6], allowing for at most two mismatches. PAR-CLIP-seq data were analysed by PARAlyzerv1.1 [7] with the recommended settings, and the T-to-C mutation was used for identification of 4SU-PAR-CLIP binding sites. DAZ-binding motifs were identified by HOMER findMotifsGenome.pl [8] from PAR-CLIP peaks of two biological replicates. Motif length was restricted to 4 - 8 nucleotides. Since polyadenylation can not only add poly A tail but also modify the length of the 3'UTR region, we aligned the polyadenylation sites (PAS, data from human cells were previously reported[9]) to the longest transcript and counted the number of UGUU motifs in the 3′UTR region upstream to PAS to investigate whether the effect of DAZ-enhanced translation is dependent on the number of binding sites in mRNAs. The Mann-Whitney U test was used to detect the difference in translation efficiency between the two groups (Group 1 (motif occurrences≤12) and Group 2 (motif occurrences>12)). Also, we chose PAS as the starting point of poly A tail and counted all of distance (number of nucleotides) between the UGUU motifs and the PAS. The correlation between distance and translation efficiency was tested using Pearson correlation coefficient.

For analysis of RIP-seq and RNA-seq data, pre-processed data were aligned to the human genome (GRC38) by HISAT2 [10] (version 2.2.1). The sorted mapping files were used as inputs for quantification with StringTie (v2.1.7) [11]. The DESeq2 package in R was used to analyse differential expressed gene [12].

For analysis of Ribo-Seq data, processed reads were aligned to the reference genome (GRCh38) by STAR (version 2.7.10a) [6], allowing for at most two mismatches. StringTie (v2.1.7) [11] was used to calculate the raw read count. Both read counts for Ribo-seq and RNA-seq were converted to TPM (transcripts per million). Genes having a threshold expression level (Ribo-Seq TPM >1 and RNA-Seq TPM >5) were further analyzed. Translational efficiency was calculated as TPM_Ribo-Seq_ / TPM_RNA-Seq_ [13] and the fold change was analysed by Limma (version 3.48.3) [14]. *P* value < 0.05, fold change > 1.5.

**REFERENCES**

1. Zhao L, Yao C, Xing X, Jing T, Li P, Zhu Z, Yang C, Zhai J, Tian R, Chen H *et al*: **Single-cell analysis of developing and azoospermia human testicles reveals central role of Sertoli cells**. *Nat Commun* 2020, **11**(1):5683.

2. Hafner M, Landthaler M, Burger L, Khorshid M, Hausser J, Berninger P, Rothballer A, Ascano M, Jr., Jungkamp AC, Munschauer M *et al*: **Transcriptome-wide identification of RNA-binding protein and microRNA target sites by PAR-CLIP**. *Cell* 2010, **141**(1):129-141.

3. Heiman M, Schaefer A, Gong S, Peterson JD, Day M, Ramsey KE, Suárez-Fariñas M, Schwarz C, Stephan DA, Surmeier DJ *et al*: **A translational profiling approach for the molecular characterization of CNS cell types**. *Cell* 2008, **135**(4):738-748.

4. Chothani SP, Adami E, Widjaja AA, Langley SR, Viswanathan S, Pua CJ, Zhihao NT, Harmston N, D’Agostino G, Whiffin N *et al*: **A high-resolution map of human RNA translation**. *Molecular Cell* 2022, **82**(15):2885-2899.e2888.

5. Langmead B, Trapnell C, Pop M, Salzberg SL: **Ultrafast and memory-efficient alignment of short DNA sequences to the human genome**. *Genome Biology* 2009, **10**(3).

6. Dobin A, Davis CA, Schlesinger F, Drenkow J, Zaleski C, Jha S, Batut P, Chaisson M, Gingeras TR: **STAR: ultrafast universal RNA-seq aligner**. *Bioinformatics* 2012, **29**(1):15-21.

7. Corcoran DL, Georgiev S, Mukherjee N, Gottwein E, Skalsky RL, Keene JD, Ohler U: **PARalyzer: definition of RNA binding sites from PAR-CLIP short-read sequence data**. *Genome Biology* 2011, **12**(8):R79.

8. Ma H, Wang X, Cai J, Dai Q, Natchiar SK, Lv R, Chen K, Lu Z, Chen H, Shi YG *et al*: **N6-Methyladenosine methyltransferase ZCCHC4 mediates ribosomal RNA methylation**. *Nature Chemical Biology* 2018, **15**(1):88-94.

9. Stroup EK, Ji Z: **Deep learning of human polyadenylation sites at nucleotide resolution reveals molecular determinants of site usage and relevance in disease**. *Nat Commun* 2023, **14**(1):7378.

10. Kim D, Paggi JM, Park C, Bennett C, Salzberg SL: **Graph-based genome alignment and genotyping with HISAT2 and HISAT-genotype**. *Nature Biotechnology* 2019, **37**(8):907-915.

11. Pertea M, Pertea GM, Antonescu CM, Chang T-C, Mendell JT, Salzberg SL: **StringTie enables improved reconstruction of a transcriptome from RNA-seq reads**. *Nature Biotechnology* 2015, **33**(3):290-295.

12. Love MI, Huber W, Anders S: **Moderated estimation of fold change and dispersion for RNA-seq data with DESeq2**. *Genome Biology* 2014, **15**(12):550.

13. Wang X, Zhao Boxuan S, Roundtree Ian A, Lu Z, Han D, Ma H, Weng X, Chen K, Shi H, He C: **N6-methyladenosine Modulates Messenger RNA Translation Efficiency**. *Cell* 2015, **161**(6):1388-1399.

14. Ritchie ME, Phipson B, Wu D, Hu Y, Law CW, Shi W, Smyth GK: **limma powers differential expression analyses for RNA-sequencing and microarray studies**. *Nucleic Acids Research* 2015, **43**(7):e47-e47.
